# Interpreting Sequence Variation in PDAC-Predisposing Genes Using a Multi-Tier Annotation Approach Performed at the Gene, Patient, and Cohort Level

**DOI:** 10.3389/fonc.2021.606820

**Published:** 2021-03-05

**Authors:** Michael T. Zimmermann, Angela J. Mathison, Tim Stodola, Douglas B. Evans, Jenica L. Abrudan, Wendy Demos, Michael Tschannen, Mohammed Aldakkak, Jennifer Geurts, Gwen Lomberk, Susan Tsai, Raul Urrutia

**Affiliations:** ^1^ Bioinformatics Research and Development Laboratory, Genomic Sciences and Precision Medicine Center, Medical College of Wisconsin, Milwaukee, WI, United States; ^2^ Clinical and Translational Sciences Institute, Medical College of Wisconsin, Milwaukee, WI, United States; ^3^ Department of Biochemistry, Medical College of Wisconsin, Milwaukee, WI, United States; ^4^ Division of Research, Department of Surgery, Medical College of Wisconsin, Milwaukee, WI, United States; ^5^ Genomic Sciences and Precision Medicine Center, Medical College of Wisconsin, Milwaukee, WI, United States; ^6^ Division of Surgical Oncology, Department of Surgery, Medical College of Wisconsin, Milwaukee, WI, United States; ^7^ LaBahn Pancreatic Cancer Program, Medical College of Wisconsin, Milwaukee, WI, United States; ^8^ Genetic Counseling Program, Medical College of Wisconsin, Milwaukee, WI, United States; ^9^ Department of Pharmacology and Toxicology, Medical College of Wisconsin, Milwaukee, WI, United States

**Keywords:** pancreatic cancer, genetic predisposition, medical oncology, precision oncology, genomic data interpretation, survival analysis, structural bioinformatics

## Abstract

We investigated germline variation in pancreatic ductal adenocarcinoma (PDAC) predisposition genes in 535 patients, using a custom-built panel and a new complementary bioinformatic approach. Our panel assessed genes belonging to DNA repair, cell cycle checkpoints, migration, and preneoplastic pancreatic conditions. Our bioinformatics approach integrated annotations of variants by using data derived from both germline and somatic references. This integrated approach with expanded evidence enabled us to consider patterns even among private mutations, supporting a functional role for certain alleles, which we believe enhances individualized medicine beyond classic gene-centric approaches. Concurrent evaluation of three levels of evidence, at the gene, sample, and cohort level, has not been previously done. Overall, we identified in PDAC patient germline samples, 12% with mutations previously observed in pancreatic cancers, 23% with mutations previously discovered by sequencing other human tumors, and 46% with mutations with germline associations to cancer. Non-polymorphic protein-coding pathogenic variants were found in 18.4% of patient samples. Moreover, among patients with metastatic PDAC, 16% carried at least one pathogenic variant, and this subgroup was found to have an improved overall survival (22.0 months versus 9.8; p=0.008) despite a higher pre-treatment CA19-9 level (p=0.02). Genetic alterations in DNA damage repair genes were associated with longer overall survival among patients who underwent resection surgery (92 months *vs.* 46; p=0.06). *ATM* alterations were associated with more frequent metastatic stage (p = 0.04) while patients with *BRCA1* or *BRCA2* alterations had improved overall survival (79 months *vs.* 39; p=0.05). We found that mutations in genes associated with chronic pancreatitis were more common in non-white patients (p<0.001) and associated with longer overall survival (52 months *vs.* 26; p=0.004), indicating the need for greater study of the relationship among these factors. More than 90% of patients were found to have variants of uncertain significance, which is higher than previously reported. Furthermore, we generated 3D models for selected mutant proteins, which suggested distinct mechanisms underlying their dysfunction, likely caused by genetic alterations. Notably, this type of information is not predictable from sequence alone, underscoring the value of structural bioinformatics to improve genomic interpretation. In conclusion, the variation in PDAC predisposition genes appears to be more extensive than anticipated. This information adds to the growing body of literature on the genomic landscape of PDAC and brings us closer to a more widespread use of precision medicine for this challenging disease.

## Introduction

Pancreatic ductal adenocarcinoma (PDAC) is a significant unsolved problem in modern healthcare (1) because most patients have metastatic disease at the time of diagnosis. Therefore, methods for risk assessment and early detection are top priorities in the quest to improve the survival of affected patients. The foundation of most early diagnosis strategies is to identify at-risk individuals through an improved understanding of genetic risk factors. Importantly, this may also allow for the development of new therapeutic targets as we define the molecular and epigenetic profile of affected patients. Recent evidence suggests that genomic variations in cancer predisposition genes are found at a higher prevalence in patients with cancer than has been previously understood (2, 3). Known pathogenic variants in these genes are rare, and most of the genetic alterations observed in patients with cancer are novel and therefore classified as variants of uncertain significance (VUS). A finding of VUS leaves both geneticists and other members of the clinical team with an inability to translate such information to a defined treatment plan and is unable to clarify cancer risk for family members. Yet, understanding of what criteria should be used in assessing VUS continues to progress. For example, studies are revealing un-recognized Mendelian causes of complex diseases (4) genetic epistasis (5) the rate of somatic variation in human blood cells (6–9), individual alleles in the same gene with different phenotypes (10), and private variants that can be pathogenic (11–13). Further, significant increases in PDAC patient survival with underlying homologous recombination deficiency (14) and molecular-targeted agents (15), combined with a high incidence of pathogenic germline variation not identified by current clinical protocols (16, 17). Thus, there is evidence that germline contributions to PDAC etiology and outcomes may be broader than currently recognized.

Previous studies examining germline contributions to PDAC have recently identified associations with previously established DNA damage repair deficiency mutations and proto-oncogenes such as KRAS (2, 3, 18, 19). However, since PDAC is uncommon in the general population (1), the complete repertoire of genomic variation that underlies this disease remains to be fully characterized. For example, most previous studies of the germline alterations in PDAC have used different small to medium-sized gene panels, making the interpretation of genetic variation across all studies difficult to compare. In addition, bioinformatics approaches used to identify and classify genomic variants differ significantly, again leaving the interpretation of genomic data unclear and inconsistent. We believe that the design of specific approaches to increase the yield of clinically relevant information is needed.

Toward this end, we designed the current study to assess germline genetic variants in a cohort of 538 patients with PDAC using a newly developed custom gene panel consisting of genes related to DNA-Damage Repair (DDR), cell checkpoint and cycle, pancreatitis and cell migration. Importantly, we employed a multi-tier approach for genomic annotation and summarized findings at the gene, patient, and cohort level. Genomic annotation is a critical step in analysis wherein the power of large database is applied to determine if variants observed in our cohort have been previously observed in others. Most commonly, germline databases are consulted for germline studies. In this study, we chose a more integrated approach by leveraging data spanning inherited disorders, germline cancer predisposition, and somatic mutations in many human tumor types. We found non-polymorphic protein-coding pathogenic variants in 18.4% of samples (27.1% when including non-coding pathogenic variants), reflecting both the incidence of known inherited cancer syndromes, as well as germline diseases with a potential contribution to PDAC. Because of differences in term use between genetics and oncology practices, we will use the terms *variant* and *mutation* somewhat interchangeably and distinguish by context; all variants in our cohort data are germline. All patient samples had a coding VUS that has been previously reported in other studies and 90% of samples carried newly described novel VUS. Moreover, using molecular models of the proteins to further investigate the VUS, we identified alteration patterns in 3D, often around specific functional sites, suggesting possible mechanisms affected by those variants. Finally, we assessed the impact of genomic sub-groups derived from our analytic approach on patient outcomes. We are optimistic that affordable custom-designed panels, when combined with a multi-tier annotation approach, will help to inform both genomic sciences and the evolving field of personalized medicine for patients with PDAC. Our results build from ideas, nascent in the field, about genomic features with mechanistic and biomedical relevance that we believe will expand the utility of precision oncology by enabling future research to increase the number of VUS that are functionally interpreted.

## Methods

### Study and Experimental Design

Patient samples included 538 isolates of peripheral blood mononuclear cells (PBMC) obtained from our PDAC biobank (whole blood) for germline DNA sequencing. This study was performed according to the Medical College of Wisconsin (MCW) IRB and used the Surgical Oncology Tissue Bank (PRO # 12151), which provided the samples, and the Pancreatic Cancer Clinical Database (PRO # 12479), which provided all clinical data. Germline DNA was isolated from cell pellets, using DNeasy blood and tissue spin columns (Qiagen), and concentrations measured fluorescently with minimum concentrations at 15ng/uL. We developed a custom gene panel targeting 53 genes (**Supplemental Table S1**) gathered for their known roles across DNA damage repair, matrix reorganization, pancreatitis risk, and others. Genes represented three groups, namely 1. genes most frequently linked to pancreatic cancer (e.g., *ATM*), 2. genes associated to preneoplastic conditions of the pancreas, and 3. genes seldom found in PDAC, but which are associated with other neoplastic and non-neoplastic diseases (2, 3, 18–21). Including the last category is important since many individuals of families carrying pathogenic variants that influence the development of cancer, often induce neoplastic transformation in other organs. Pathobiologically, these genes belong to the following ontological categories: DNA damage response (n=19), cell cycle regulators and checkpoints (n=17), pancreatitis (n=5), and cell adhesion or migration (n=6). Several genes have overlapping functions, such as *TP53* and *ATM*, which are involved both cell cycle and DNA damage sensing. Thus, our testing platform is inexpensive and informative, two key characteristics that can make this tool widely used in clinical practice. Amplicons were designed with Illumina’s custom panel software using the GRCh37 assembly. We called this panel the MCW PDAC Germline AmpliSeq Panel. Library preparation and sequencing was completed at MCW’s Genomic Science and Precision Medicine Center (GSPMC). Briefly, 10 ng of DNA input for each of two primer pools was aliquoted and prepared using the AmpliSeq for Custom panels kit (Illumina) with 16 cycles of gene specific amplification. Targets were enzymatically digested before ligation of sample specific indexes and library clean up. Prepared libraries were then amplified an additional seven cycles, cleaned up with AMPure XP beads (Beckman Coulter, Life Sciences) and assessment of library quality completed with the Fragment Analyzer (Agilent, Standard Sensitivity NGS fragment Analysis). Quantified libraries were then pooled (with 56–77 samples per each of eight pools) and paired-end sequenced on the Illumina MiSeq platform at 2x300bp. Sequencing was performed with a median target coverage of 309 reads (**Supplemental Table S1**).

We generated quality metrics at all stages in the process, including sample, library, flowcell, sequence mapping, and read and base quality (**Supplemental Text**). Data was processed using our bioinformatics workflow build using GATK v3.7 and HaplotypeCaller (22).

### Annotation of Genomic Variants

To interpret genomic variation, and as is common in the field (23), we gathered existing annotations from national consortia resources. However, we took a more integrated approach than is often used and leveraged across the germline resources of Human Gene Mutation Database (HGMD, 2018.1) (24), ClinVar (2018.05) (25), and Genome Aggregation Database (gnomAD, r2.0.2) (26, 27), and cancer resources of Catalogue Of Somatic Mutations In Cancer (COSMIC, v85) (28, 29), and The Cancer Genome Atlas (TCGA, mc3.v0.2.8 public mutation calls) (30). We used the BioR toolkit (v5.0.0) (31), to merge these resources and annotate our cohort. All analyses were made on genome version GRCh37. We used snpEff (v4.3i) (29) and ensemble canonical transcripts (GRCh37.75) to annotate and predict the effect of each variant on protein coding sequences. Protein-coding variants were defined as those resulting in amino acid substitution, in-frame deletions or insertions, frameshifts, splice donor and acceptor alterations, and start/stop codon alterations. The remaining variant types were considered non-coding. Database annotations were used to categorize variants according to their clinical significance (**Supplemental Figure S1**). Each variant was annotated with the germline databases, ClinVar and HGMD, and with somatic variant databases of cancer, COSMIC and TCGA. All variants were classified in one of six categories: pathogenic, benign, reported VUS, novel VUS, somatic VUS, and other. We defined pathogenic variants as those with no conflicting classifications between HGMD and ClinVar, and that were identified as (likely) disease causing mutation (DM/DM)?, disease-associated polymorphisms with supporting functional evidence (DFP), or (likely) pathogenic. We defined benign variants as (likely) benign variants without other conflicting annotation. We defined variants as VUS if there were conflicting annotations within or between HGMD and ClinVar, if they had only an explicit annotation of VUS, or if annotated as functional polymorphism (FP) or disease associated polymorphism (DP) in HGMD. We classified a variant’s significance as “other” when the only annotation was as a risk allele or a drug response phenotype. HGMD retired records (R) were ignored. Because pathogenicity is meant to describe the effect of a variant in germline disease, variants observed somatically have not been assessed in the same way as those observed in inherited disorders. For that reason, variants that have only been observed in somatic databases were classified as somatic VUS and are listed in **Supplemental Table S2**. Finally, variants with no annotation in any of the databases considered (HGMD, ClinVar, COSMIC or TCGA), were designated as novel VUS. We re-coded variant classes from ClinVar and HGMD to use the same terms of pathogenic, VUS and benign. The protein-based scores generated by CADD, Revel, SIFT, Fathmm, Lrt and Metalr were converted to the scale Deleterious, Tolerated or Not Generated (NG). The thresholds for deleterious scores were set at each tool’s default values, with the exceptions of CADD (score ≥ 30, corresponding to the most damaging 0.1% of variants) and Revel (score > 0.5). We defined functional gene sets for use in annotating our gene panel and interpreting the potential effect of variants in our cohort. DNA damage response pathways were defined as specified by The Cancer Genome Atlas Analysis Working Group (32). We defined cell cycle or cycling checkpoints by a combination of Reactome (G2/M checkpoints, cell cycle checkpoints, and regulation of mitotic cell cycle), KEGG (cell cycle), and BioCarta pathways (cell cycle), augmented by the results from Fischer (33) and Whitfield (34); we used these pathways as organized by MSigDB (35). There is a known association between chronic pancreatitis and development of pancreatic cancer (36), but varying reports on the outcomes associated with chronic pancreatitis among PDAC patients (37, 38). Thus, to investigate potential associations with germline genetic factors related to PDAC and CP, we defined a chronic pancreatitis genetic pathway by alterations of *CFTR*, *CTRC*, *CPA1*, *SDHA*, *SDHB*, *SDHC*, or *SDHD*. SDH subunits were considered due to the indirect link to HIF1a regulation and pseudohypoxia that has growing evidence supporting (39–41).

### Calculations of Allele Enrichment and Depletion

For each variant, we identified its minor allele frequency (MAF) within our cohort and in the currently healthy adult population (gnomAD) (26). The ratio of these two MAFs is an estimation of the enrichment or depletion of the variant, which may be a signal for new risk or protective alleles, respectively. We will refer to variants as “enriched” or “depleted”, according to MAF ratio in our cohort relative to gnomAD. We defined polymorphic variants as those with a MAF of at least 5% in gnomAD. We defined “private” variants as those seen only in one patient sample. We used Fisher’s Exact test to compare the number of alleles for a given variant observed in our cohort compared to the same in gnomAD and report the one-sided test for a greater occurrence in our cohort.

### Molecular Modeling and Structural Bioinformatics

We modeled five select genes as their protein products in 3D, to facilitate interpretation of the observed genomic variants. We used homology-based methods (42, 43) combined with review of the models for quality control (44) and integration with electron microscopy density maps, when available. To model ATM, we utilized the 5.7Å electron microscopy map of human dimeric ATM deposited in the PDB (45) [reconstructed in 5np0 (46)]. To model the MutSα complex, we used the 2.75Å crystal structure of ADP-bound human MSH2/MSH6 heterodimer [2o8b (47)]. The PALB2 model was built from the 1.9Å crystal structure of the C-terminal WD40 domain of human PALB2 [2w18 (48)] and the CHEK2 model from the 3.0Å crystal structure of the human dimeric CHEK2 [3i6u (49)]. The POLE models were based on the N-terminal 2.2Å crystal structure of *Saccharomyces cerevisiae* DNA Pol epsilon [4m8o (50)] and the C-terminal 4.98Å electron microscopy map of *Saccharomyces cerevisiae* DNA Pol epsilon [reconstructed in 6hv9 (51)] along with human POLE sequence to create human homology model structures. Each human homology model structure was created with Discovery Studio’s Create Homology Models tool (Biovia, v19.1.0.18287). The models were annotated with domains as identified in Pfam 32.0 (52) accessed through neXtProt (53). Variants were mapped to our 3D models using custom scripts and visualized using PyMOL version 1.9.0 (54).

### Clinical Outcomes Association Analysis

Using a prospectively maintained pancreatic cancer database at MCW, we reviewed consecutive patients with biopsy-proven PDAC from 2009 to 2017. Statistics were performed as previously described (55, 56). Briefly, categorical variables were compared using Chi-squared test or Fischer’s Exact test. Continuous variables were analyzed using the Mann-Whitney U test. Survival and follow-up were calculated from the time of initial diagnosis to the date of death or last follow-up, with deaths from any cause included in the survival analysis. Overall survival was estimated using the Kaplan–Meier method and the log-rank test was used to compare survival distributions between groups. Survival and outcomes analyses were performed using Stata 13.1 (StataCorp, College Station, TX, USA).

## Results

### Identification of a High Level of Genomic Variation in PDAC-Predisposition Genes in a Referral Population with Confirmed Diagnosis of Pancreatic Cancer

**Figure 1:**
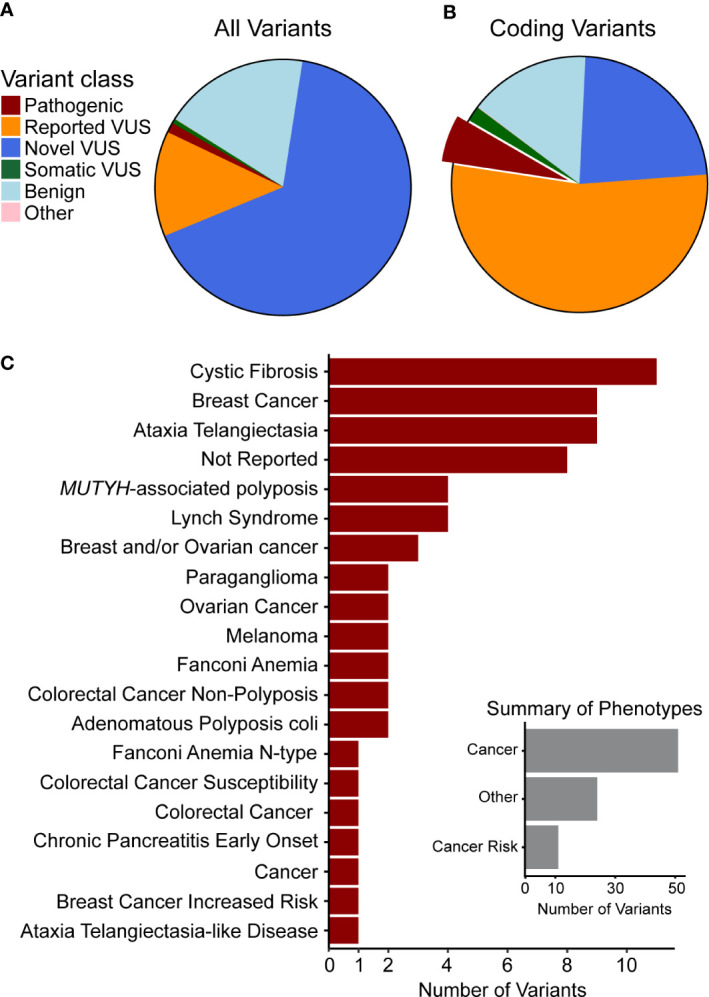
Genetic variants observed in our cohort are novel or have been previously associated with cancer. Clinical significance of variants was synthesized from data aggregation (see *Methods*) with discrepancies and disease context (germline or somatic) summarized as different types of VUS (**Supplemental Figure 1**). **(A)** Classification of all variants and **(B)** of coding variants shows the majority to be VUS. The portion of pathogenic variants is highlighted and those variants are used in the following summary. **(C)** The top 20 phenotypes associated with previously observed germline pathogenic variants indicates that many are related to cancer or cancer predisposition. Thus, we curated the phenotypes (Inset) to quantify this relationship and found that most are directly associated with cancer or with cancer risk.

For the identification, annotation, classification, and phenotypic association of known and novel variants in PDAC-predispositions genes, we sequenced DNA from peripheral blood monocyte cells obtained from a cohort of 538 patients with histologically confirmed PDAC (**Table 1**). Sequencing each sample at a median depth of 309.2x (± 88.5) identified a total of 5,961 variants, comprised of 4,968 non-coding (83%) and 993 (17%) coding alterations (**Table 2**, **Supplemental Tables S1, S3**), constituting both previously described and a large percentage of novel variants. For interpreting this extensive variation, we tested an enhanced annotation approach wherein we combined information derived from inherited disorders (ClinVar and HGMD) and from studying somatic variation (TCGA and COSMIC). We categorized the variants found in our cohort as pathogenic, benign, previously reported variant of unknown significance (VUS), novel VUS, somatic VUS, or other (see *Methods*; **Supplemental Figure S1**). This classification yielded 75 known pathogenic variants, which represent 1.3% of the all the variants reported herein, in 25 of the 53 genes tested. Five of these 75 variants are polymorphic, reducing but not eliminating their likelihood of disease relevance; we reported polymorphic variants but excluded them for most of our analyses. The remaining 70 were observed in 27.1% of patient samples. Notably, we also discovered a significant number of VUS, comprising 4780 (80.2%) of the total (**Figure 1A**) and 780 (78.5%) of coding variants (**Figure 1B**). The majority of VUS affecting coding regions were previously reported (53.6%) and missense or frameshift, while non-coding variants were largely novel VUS (74.9%) and intronic (including splice sites) or synonymous (**Supplemental Table S4**). We quantified the ratio of non-synonymous to synonymous variants at the cohort (0.99) and per-patient level (0.49 ± 0.08). This difference in the ratio between the cohort and average across patients indicates the relative pressure against coding variation in these genes and underscores the importance of new methods, such as we use below, to interpret private missense mutations. Thus, we observed a large set of novel variants, many of which are protein coding and private, motivating a need for more integrated approaches for evaluating their potential disease and clinical relevance.

**Table 1:** Demographics of patients in the cohort.

Patients Enrolled	n = 538
Age, years median (IQR)	65 (13)
Gender, n (%)	
Female	252 (47)
Male	286 (53)
Race, n (%)	
White	477 (89)
Black	35 (7)
Hispanic	12 (2)
Asian	7 (1)
Native American/Alaskan	2 (0)
Other	5 (1)
Radiographic Stage, n (%)	
Resectable	165 (31)
Borderline	180 (33)
Locally Advanced	109 (20)
Metastatic	84 (16)
Pre-treatment CEA, ng/ml median (IQR)	3.5 (4.6)
CA19-9 producers, n (%)	303 (69)
Pre-treatment CA19-9, U/ml median (IQR)	321 (838)
Post-treatment CA19-9, U/ml median (IQR)	53 (132)
Post-surgery CA19-9, U/ml median (IQR)	19 (28)
	
**Patients with Survival Data **	n = 461
Vital Status (Dead), n (%)	319 (69)
Median Overall Survival – All patients, months (n)	
Resectable	45.1 (n=154)
Borderline	27.2 (n=164)
Locally Advanced	29.1 (n=81)
Metastatic	10.6 (n=62)
Median Overall Survival – Resected patients, months	
Resectable	55.1 (n=137)
Borderline	38.2 (n=112)
Locally Advanced	42.4 (n=37)

**Table 2:** Distribution of variant classification based on their potential impact on the protein sequence.

	Non-coding	Coding	% Coding
Pathogenic	16	59	78.7%
Reported VUS	261	532	67.1%
Novel VUS	3724	229	5.8%
Somatic VUS	15	19	55.9%
Benign	948	153	13.9%
Other	4	1	20.0%
**TOTAL**	**4,968**	**993**	**16.7%**

We hypothesized that the pathogenic variants we observed in our cohort could contribute to disease risk and underlying but un-recognized familial PDAC (FPC), or *de novo* occurrence of risk alleles. We were able to complete records review for all patients with samples containing pathogenic variants and found that 33 (23%) had a personal history of cancer. Thus, the incidence of FPC is likely even higher than 23% in our cohort. Compared to the national average of 10% FPC (57), it is likely that some of these alleles represent un-recognized cases of FPC.

Pathogenic variants observed in our cohort (**Supplemental Table S5**) affected DNA damage repair genes that have known association with cancer risk in the pancreas and other organs. Truncating and frameshift loss-of-function (LoF) changes account for 47 of these alterations, while 12 were missense, and 16 were non-coding. LoF alleles were most common in *ATM* (14 variants), *BRCA2* (6), *PALB2* (4), and *BRCA1* (3), as well as *CFTR* (6), which is linked to the development of chronic pancreatitis, a disease that associates with pancreatic cancer development (2, 3, 18–21). In addition, we found missense variants primarily affecting *CFTR* (4 variants) and *MUTYH* (2). The non-coding variants mainly affected DNA repair genes, such as *MUTYH* (four variants), *PMS2* (2), and *MHS6* (2). Moreover, we correlated the pathogenic variants identified in our cohort with the phenotypes reported from previous cases, which showed that most of them were directly associated with cancer or cancer risk (**Figure 1C**, inset). We found that 10 of the pathogenic variants were associated to breast cancer, 11 to cystic fibrosis, and 9 to ataxia telangiectasia (**Figure 1C**), further supporting their relevance to patient disease in our cohort. Additional mutations have been previously reported from other cancer types, but they affect DNA damage sensing and repair pathways, which is a pan-cancer mechanism that is clearly relevant to PDAC. Finding these variants highlights our current incomplete understanding of the interplay between germline and somatic disease mechanisms, specifically the potential interplay of growth and differentiation pathways that are operational in cancer development.

A subset of nine pathogenic variants that have previously been associated with different forms of pancreatic cancer (six variants) and pancreatitis (three variants) affected 5.4% of the cohort (29 patients, **Table 3**). All of these variants had a frequency ratio >1.9 in our cohort as compared to gnomAD, and four with p < 0.01, indicating a potential enrichment in our cohort and thus a potential contribution from these alleles in PDAC development or progression (**Table 3**). All variants with p > 0.01 were private in our cohort and rare in gnomAD, which does not necessarily preclude their contribution to rare diseases, such as pancreatic cancer, with a later age of onset (58, 59). Of the six pancreatic cancer variants associated with an elevated cancer risk, five were private to individual patients, and the variant in *TP53* (p.Arg248Gln) was identified in two individuals. Two rare population variants in *BRCA2* (< 0.026% MAF) and two in *BRCA1* (0.02% MAF) were reported in our cohort (**Table 3**). We also identified rare variants in *PALB2* and *TP53*. The c.743G>A, p.R248G in *TP53* has been associated with multiple phenotypes, including pancreatic adenocarcinoma. Interestingly, other studies of PDAC have reported two additional variants with different amino acid substitutions at the same site as p.R248G, supporting its relevance in our cohort (60–62). In summary, these genetic variants have a direct mechanistic role in PDAC, and as germline mutations they should be integrated into the evolving field of precision medicine.

**Table 3:** Pathogenic variants in genes conferring pancreatic cancer or pancreatitis susceptibility.

Gene	HGVSc	HGVSp	Variant ID	ClinVar or HGMD Phenotype	Patient Number	Population Frequency	Cohort Frequency	Frequency Ratio	Fischer’s Exact Test p-value†
*BRCA1*	c.5329dupC	p.Gln1777fs	rs80357906	Pancreatic cancer susceptibility	1	1.62E-04	9.29E-04	5.73	1.6E-01
*BRCA1*	c.68_69delAG	p.Glu23fs	rs80357410	Pancreatic cancer susceptibility	1	1.99E-04	9.29E-04	4.68	2.0E-01
*BRCA2*	c.5682C>G	p.Tyr1894*	rs41293497	Pancreatic cancer susceptibility	1	4.08E-06	9.29E-04	227.96	8.7E-03
*BRCA2*	c.5946delT	p.Ser1982fs	rs80359550	Pancreatic cancer susceptibility	1	2.60E-04	9.29E-04	3.58	2.5E-01
*PALB2*	c.509_510delGA	p.Arg170fs	rs863224790	Pancreatic cancer susceptibility	1	3.25E-05	9.29E-04	28.60	1.8E-01
*TP53*	c.743G>A	p.Arg248Gln	rs11540652	Pancreatic adenocarcinoma	2	2.03E-05	1.86E-03	91.56	1.5E-03
*CFTR*	c.1652G>A		rs75527207	Cystic fibrosis, Hereditary pancreatitis	1	1.84E-04	9.29E-04	5.04	5.7E-03
*CTRC*	c.*86A>G		rs760937	Pancreatitis	17	9.76E-03	1.90E-02	1.95	3.8E-02
*CPA1*	c.79C>T	p.Arg27*	rs141209213	Pancreatitis	4	4.35E-04	3.72E-03	8.54	3.9E-04

HGVS, Human Genome Variation Society.

^†^We used the 2x2 test to compare the number of alleles of each variant in our cohort compared to gnomAD. Asterisks indicate transcript termination via stop codon, or alteration of a stop codon.

Subsequently, we evaluated whether our subjects carried mutations that supported the pancreatitis-to-pancreatic cancer evolution hypothesis (63). Interestingly, all three pancreatitis pathogenic variants examined were more prevalent in our cohort than the general population, two were associated with non-hereditary pancreatitis that included chronic, early onset pancreatitis (*CPA1*, ratio of 8.54) and chronic pancreatitis (*CTRC*, ratio of 1.95), while one variant was associated with hereditary pancreatitis (*CFTR*, ratio of 5.04). Expanding our view to all variants found in *CFTR*, we observed 23 coding VUS in 393 patients (73% of the cohort), and 11 pathogenic coding variants (**Supplemental Table S5**) in 28 individuals (5.2% of the cohort). Of these coding variants, 22 were rarely observed in the normal healthy population, but affected 76 patients (14.1%) in our cohort. The c.815T>C and c.4129G>C variants represent a novel and known VUS, respectively, and were seen in one patient each but absent from gnomAD. Two *CFTR* coding pathogenic variants [c.1521_1523delCTT (p.Phe508del) and c.3909C>G (p.Asn1303Lys)], were recurrent in our cohort, found in 16 and three patients, respectively (**Supplemental Table S5**). Both variants are associated with cystic fibrosis in HGMD. Overall, we identified 16 coding variants with potential clinical impact (pathogenic, reported VUS or novel VUS) that were detected in more than one patient in our cohort (**Supplemental Table S6**); three were absent in gnomAD and a further six had p<0.01 for enrichment. For these reasons, we propose that these *CTFR* variants, with the potential to modulate lung and pancreatic mucosal functions, should be considered for further validation studies to understand their disease or response associations, as well as underlying mechanisms beyond the simplification of associating them with chronic pancreatitis.

Of note, chronic pancreatitis has an incidence of 50/100,000 people (36, 64), which is less than the rate of pathogenic pancreatitis alleles observed in our cohort. The *CTRC* pancreatitis pathogenic allele (c.*86A>G) (24) was detected in 17 (3.2%) patients and is less common in the healthy population (0.98% MAF; **Supplemental Table S5**). A *CPA1* pathogenic pancreatitis allele was identified in 4 patients (0.74%) but is rare in the healthy population (0.044% MAF with no homozygous individuals). These genetic mutations are likely to play a role in pancreatic cancer by increasing the probability of the tissue to become dysregulated in function, altered in morphology, and inflamed (65). The increased frequency of pancreatitis-associated variants in our cohort of patients with confirmed PDAC compared to the currently healthy adult population supports this view. Thus, this data should alert physicians to the fact that some patients with pancreatic cancer may have suffered from an unsuspected, mild form of chronic pancreatitis which may predispose to an increased risk for PDAC – an event which could potentially be modulated by alleles predisposing to either disease.

### Novel VUS in PDAC-Predisposition Genes Provides Evidence of Potentially Under-Recognized Mechanisms of Disease

Our analyses underscore that many of the protein-coding genetic variants observed in our study have neither been reported in germline nor in somatic genomic disease databases, making them novel VUS (229 of 993 variants, 23.1%, **Table 2**, **Figure 1B**) and requiring separate, further considerations. Since our study was designed to focus on genes with relevance to PDAC predisposition, it can identify novel variants with potentially unrecognized contributions to risk. At the gene level, *BRCA2*, *POLE*, *NF1*, *ATM*, and *CFTR* had the most novel coding VUS (**Figure 3C**). However, when we also considered non-coding novel VUS, we observed the same affected genes, plus additional genes, such as *TSC2*, *SMARCA4*, and *POLD1* (**Supplemental Figure 3C**). The importance of these observations is that they indicate the potential contributions of the affected genes to DNA Damage Repair and epigenetic regulation, with the latter an increasingly recognized mechanism linked to pancreatic cancer.

We subsequently tested the idea that patients with PDAC may be enriched for novel or rare alleles in their germline, which would indicate potential disease predisposition (26). First, we investigated the 174 (76%) novel coding VUS that have never been observed in the healthy population. Within our cohort, 95 of them were seen in a single sample, 79 in at least two samples, and 10 in more than 10 samples. These 10 alleles were in *CPA1* (3 variants), *BRCA2* (2), *PTEN*, *SDHD*, *MRE11A*, *NF1*, and *MSH2*. Thus, we inferred that their recurrence in our cohort and absence in the reference healthy population render these variants of interest for future mechanistic experimentation using cell and animal models. Second, 55 novel coding VUS that have been observed in the healthy population were either seen more frequently than expected (enriched) or less frequently than expected (depleted) in our cohort. Of them, 53 were rare (**Supplemental Figure S2A**) and two were polymorphic. Among the 53, 48 were 2-fold enriched and 37 10-fold enriched (**Supplemental Figure S2B**). These data suggest the possibility for an unrecognized genetic contribution to PDAC risk, due to the concentration of these alleles that we found in affected individuals. These alleles were spread across many genes with *FANCG* (five variants) as the most frequently altered gene, followed by *BUB1B* (4) and *PALLD* (4). Only one allele in *PDGFRA* (c.3123-69_3123-68insG) was observed as 2-fold depleted (**Supplemental Figures S2A, B**), suggestive of a protective or neutral association to the disease. In summary, we identified a larger set of novel variants, which indicate even broader genomic variation in the tested genes than previously described (2, 3), an observation of clear relevance to the field of pancreatic cancer genomics.

## Integration of Germline and Somatic Annotations at the Patient and Cohort Level Identify Recurrent Genetic Associations with Somatic Diseases

**Figure 2:**
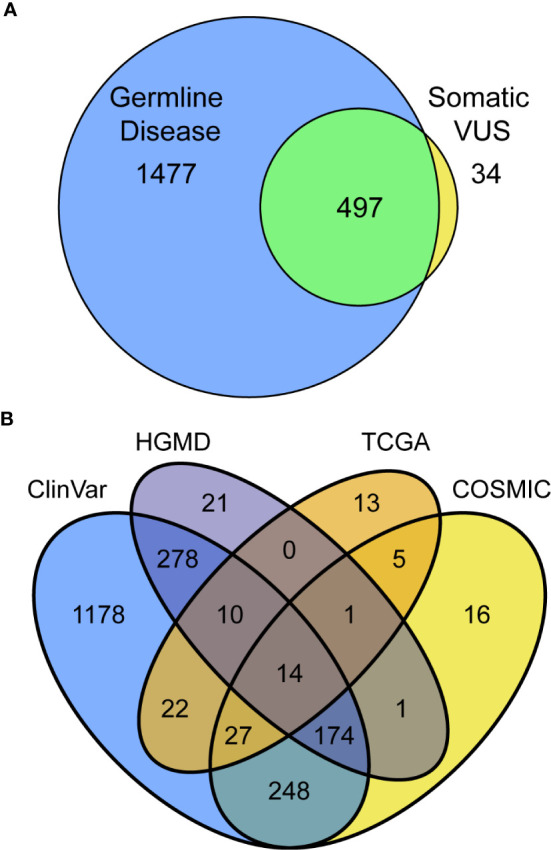
Utilizing multiple databases, germline, and somatic for variant classification increases assignments and confidence. **(A)** Intersection and separation of germline and somatic classifications for all 2,008 variants in the PDAC panel which have been previously reported. **(B)** Overlapping classifications among the germline (ClinVar and HGMD) and somatic (TCGA and COSMIC) databases shown as Venn diagram.

Thus far, we have assessed information in the way that is most commonly practiced in germline clinical genomics workflows, by looking for associations among germline resources. We next extended our assessment by considering, for the same variants, what level of evidence exists in somatic databases to better infer their likelihood of a variant being dysfunctional. This approach included variants that have only been observed in somatic disease, which otherwise would be missed by standard germline studies. Cross-referencing among germline and somatic databases identified 2008 (33.7%) previously reported variants, 34 of which have only been reported somatically and primarily in *CPA1* (five variants), *POLD1* (3), and *PDGFA* (3; **Figure 2A**, **Supplemental Table S2**). Among the 497 previously reported variants in both germline and somatic contexts, the most commonly affected genes were *ATM* (43 variants; **Supplemental Table S7**), *BRCA2* [(31); **Supplemental Table S8**), *TSC2* (26), and *APC* (23). *ATM* variants consisted of four absent from gnomAD, five rare and with p<0.01, 21 rare, and six commonly observed in gnomAD. *BRCA2* variants consisted of 14 absent from gnomAD, one rare and with p<0.01, 17 rare, and six commonly observed in gnomAD. Looking specifically at the 1974 variants previously reported in germline, 98.8%, were found in ClinVar, 25.3% in both ClinVar and HGMD, and 1.1% only in HGMD (**Figure 2B**). Among variants annotated by somatic databases, 439 were reported in COSMIC, 45 additional by TCGA, and 47 by both (**Figure 2B**). Genetic variants observed in COSMIC and detected in our cohort were frequently associated with hematopoietic and lymphoid neoplasms (79 variants), as well as carcinomas of the large intestine (66), breast (27), and lung (22). Variants detected in our cohort that had been observed in TCGA were found in endometrial cancer (43), stomach (10), and colon (10). Thus, we conclude that the extent of cancer genomic data shared across body sites, and likely relevant to PDAC, is broader than expected. Finally, we observed that 27.6% of annotated variants are in two databases, 10.6% in three, and 0.7% in all four. Thus, combined, these results demonstrate that considerable information can be gained by genomic annotation using both germline- and cancer-derived data, likely improving variant interpretation.

### Private Variants Are Highly Represented in DNA Damage Repair Enzyme Genes

**Figure 3:**
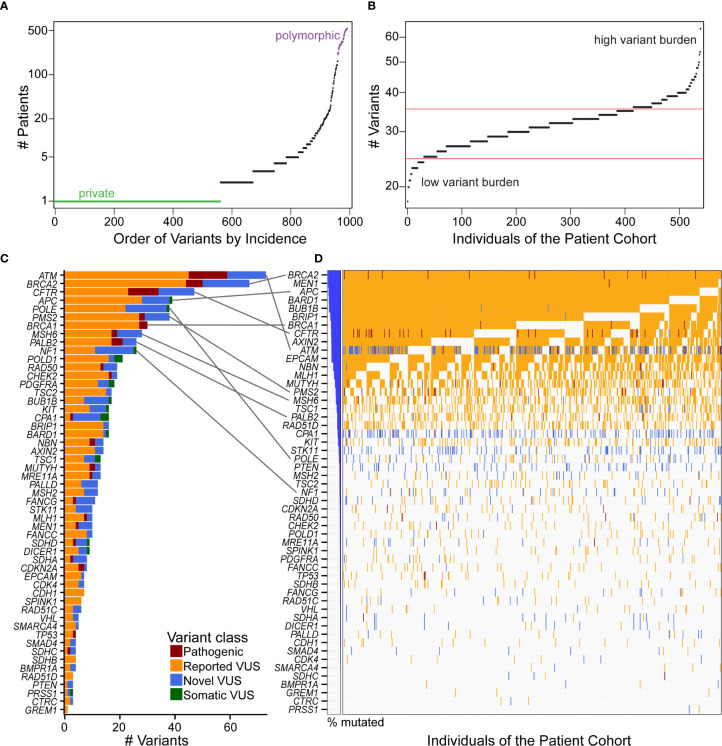
Distribution and quantification of coding variants in the patient cohort illustrates that gene with the most variants does not correspond to the highest incidence in the patient cohort. **(A)** Ordering the 993 coding variants by incidence across our cohort illustrates that 56.7% of the mutations are private (green), and there is a continuous distribution up to polymorphic (purple) alleles. **(B)** Mutational burden was fairly consistent across individuals of the cohort, with the majority of patients having 25–35 coding variants (between red boundaries) from our 53 gene panel and few patients residing in the high or low variant burden tails. **(C)** The genes with the most unique coding variants were ATM, BRCA2, and CFTR, with many genes harboring numerous pathogenic variants and VUS. **(D)** Incidence of variants across the cohort showed a different order of genes [see gray connections for top 10 genes in **(C)**]. That is, because many of the VUS are private, genes like ATM that have many unique variants are not the most frequently altered across the cohort. Interestingly, every patient has a BRCA2 variant, nearly all of which are previously reported VUS. When multiple distinct variants are present in the same gene and the same patient, the more severe class was indicated in the order of pathogenic, reported VUS, and then novel VUS.

We tested the hypothesis that there is an under-recognized prevalence of germline contributions to PDAC than currently acknowledged. To consider this premise, we investigated patterns of incidence across individuals and genes. Most coding variants (56.7%) were private, occurring in only one patient, while 20.8% were observed in at least five patient samples, with a continuous distribution of incidence (**Figure 3A**). Across patients, the distribution of mutational burden, as calculated from our cohort, showed a relatively symmetric distribution with a short upper tail, suggestive of a small number of patients who have many germline mutations (**Figure 3B**). We observed a median of 32 variants per patient. The genes with the highest overall mutational burden, considering coding and non-coding variants, were *POLE* (322 variants), followed by *NF1* (300) and *ATM* (247) (**Supplemental Figure S3C**). Considering only coding variants, the genes with highest mutational burden were *ATM* (77 variants), *BRCA2* (67), *CFTR* (47), *APC* (39), and *POLE* (38) (**Figure 3C**).

Because alleles may be recurrent across patients, the number of patients affected by these alterations was not highly correlated with the number of unique variants in each gene (**Figures 3C, D**). For example, all of our patients had a either a pathogenic variant or VUS in *BRCA2*, and nearly all in *MEN1, APC*, and *BARD1*, but only *BRCA2* and *APC* were in the top five genes for total number of variants per gene. *ATM* had the most unique variants, with 14 novel VUS of the 77 (18%) reported here. However, *ATM* was the 10^th^ most frequently observed across patients. Likewise, *POLE* had 38 variants, but was 23^rd^ in frequency across patients (**Figures 3C, D**). Thus, this data indicates that a larger percentage of individuals, who present to our practice with a confirmed diagnosis of PDAC, may carry a disease-relevant genetic variant than previously anticipated (2, 3).

Due to its prominence and clinical relevance, we made a focused analysis of *BRCA2*. *BRCA2* ranked second in number of distinct coding variants per gene (**Figure 3C**), in which we found that every patient in our cohort carried a genetic variation (**Figure 3D**). In total, we identified 210 variants in *BRCA2* with 76 affecting coding and 134 involving non-coding regions. Among coding variants, 6 of them were pathogenic (**Supplemental Table S5**), 44 reported VUS and 17 novel VUS. Breast cancer was the top phenotype associated with the previously reported variants in this gene (**Supplemental Table S5**). Of the pathogenic *BRCA2* variants, we observed that three of the six have a frequency ratio greater than 40, indicating a significant enrichment in our cohort compared to the general population. Two pathogenic coding variants in this gene, c.2957delA (p.Asn986fs) and c.3847_3848delGT (p.Val1283fs), were seen in six and two patients, respectively (**Supplemental Table S5**). These variants are rare in the general population with c.2957delA (p.Asn986fs) not even present in gnomAD and c.3847_3848delGT (p.Val1283fs) showing a frequency ratio of 43.23 (**Supplemental Table S5**). Both variants have previously been reported with breast and ovarian cancer phenotypes (25). Lastly, we found that 38 coding variants with potential clinical impact (pathogenic, VUS or novel) were seen in more than one patient in our cohort (**Supplemental Table S8**). Hence, this information agrees with, but also enriches the type of annotations that extends the interpretation of genomic variation in *BRCA2*.

### Individual Patients Carry Multiple Variants in the Same Gene

**Figure 4:**
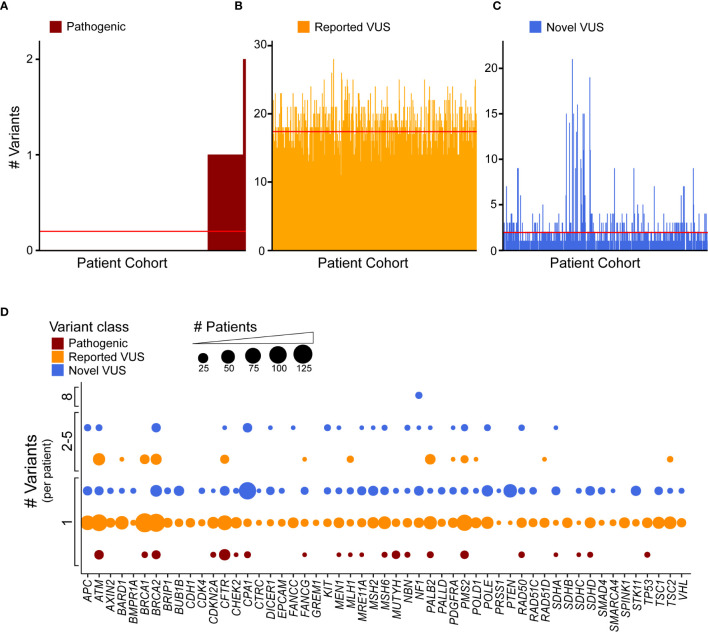
Discovery recurrent co-occurrence of genes with multiple variants in the same sample. **(A)** Number of pathogenic variants identified in each patient of the cohort with 92 patients having one pathogenic variant and 7 with two. The number of **(B)** reported VUS and **(C)** novel VUS identified in each patient. Lines indicates average number of variants across the cohort (<1 for pathogenic, 17.3 for reported VUS, 1.9 for novel VUS). The patient cohort was first sorted by the number of pathogenic variants and then reported and novel VUS graphed in the same patient order. **(D)** A bubble plot representing the number of coding variants that occur per patient, per gene. The bubble size is proportional to the number of patients that have single or multiple variant(s) in a particular gene.

Clinical genomics has focused on determining the effect of individual genetic mutations, but the process of understanding the effects of multiple variants co-occurring in the same gene, for the same patient, has not been developed. We sought to understand how frequently multiple variants co-occur in the same gene, accounting for the type of alteration. First, we identified how many patients were affected by the coding variants. Across our cohort, 99 patients (18.4%) had at least one pathogenic variant, with a subset of seven patients (1.3%) having two (**Figure 4A**). Previously reported coding VUS averaged at 17 per patient (**Figure 4B**) with the novel coding VUS averaging much lower at 1.9 (**Figure 4C**). Next, we considered the same data, but used a gene-centric perspective. We found that pathogenic coding variants occurred in 21 genes (**Figure 4D**), with nine genes (*ATM, BRCA2, CFTR, CPA1, MUTYH, PMS2, RAD50, SDHD*, and *TP53*) displaying a coding pathogenic variant observed in at least two patients. We did observe multiple VUS within the same gene and for the same patient. For instance, among previously reported coding VUS in *ATM*, we identified 27 patient samples with two concurrent variants, six with three, two with four, and one with five (**Supplemental Figure 4A**). *BRCA1, BRCA2*, and *PALB2* had similar numbers of patients with at least two previously reported coding VUS, with 17, 26, and 22 patients, respectively. Among novel coding VUS, *ATM* had 4 patients with at least two variants, 14 in *BRCA2*, 1 in *PALB2*, and none in *BRCA1* (**Supplemental Figure 4B**). Additional genes with at least two concurrent variants in multiple patients included *APC*, *NF1*, and *PMS2*. The most extreme instance occurred in five patients, who each had eight novel VUS in *NF1*. Thus, taken together, these findings suggest that the development of polygenic risk scores for PDAC is necessary to maximize the interpretation of each patient’s data; inferring how co-occurrence of multiple variants alters gene function remains unknown.

### Structural Bioinformatics Aids Interpretation and Mechanistic Insight for Genomic Variants Found in PDAC-Predisposition Genes

**Figure 5:**
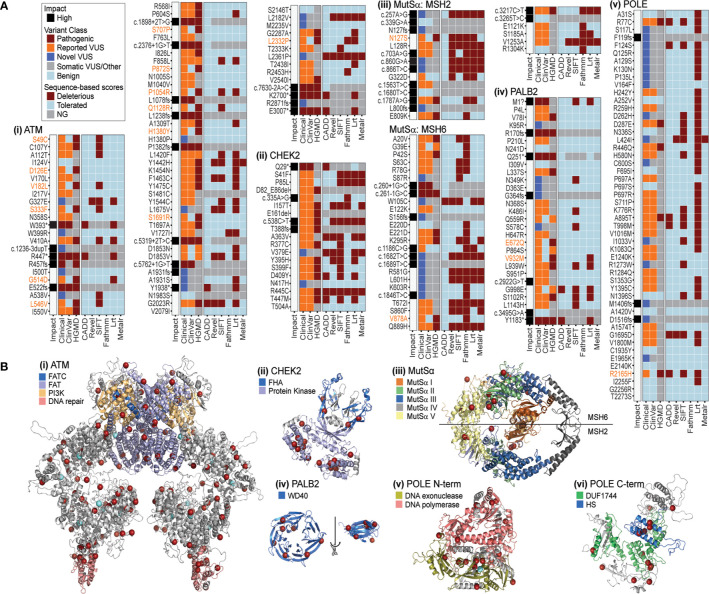
PDAC-associated genomic variation has inconsistent patterns in sequence based, but greater consistency in 3D. **(A)** Impact, variant class, and sequence-based scores for PDAC variants of (i) ATM, ii. CHEK2, iii. MutSα, iv. PALB2, v. POLE. Variants with a mismatch in the ClinVar and HGMD classification (pathogenic versus benign) are highlighted in orange text. Sequence based scores not generated (NG) are indicated in gray. **(B)** Mapping PDAC variants on the structures of (i) ATM, ii. CHEK2, iii. MutSα, iv. PALB2, v. POLE N-terminal, vi. POLE C-terminal. Red spheres mark amino acids with variants found in this PDAC study. For ATM and POLE N-terminal light blue spheres mark PDAC variants additionally reported in 5 or more samples in COSMIC. Various domains are indicated by color for each structure. FATC, FRAP-ATM-TRRAP-C-terminal; FAT, FRAP-ATM-TRRAP; PI3K, phosphoinositide 3-kinase; FHA, forkhead-associated; WD40, beta-transducin repeat; DUF1744: domain of unknown function; HS, Homo sapiens homology region.

Most alleles are of uncertain significance, even among PDAC predisposition genes with well-understood cancer-relevant functions. We chose six loci that were frequently altered in our cohort, namely *ATM, CHEK2, MSH2, MSH6, PALB2*, and *POLE*, to compare among sequence- and 3D-based methods for interpreting the likely underlying mechanisms for, and thereby disease relevance of, the genetic changes observed in our cohort. We refer to the combination of DNA sequence-based scores, used by most laboratories, as sequence-based methods of variant interpretation. We denote the use of computational biophysics, biochemistry, and structural bioinformatics, as 3D methods of variant interpretation. Previous studies from our laboratory, applying these methods to monogenic diseases, have shown that their performance is most often superior to sequence-based techniques (68, 69) – in particular, for identifying underlying mechanisms of protein dysfunction. We tested the utility of 3D protein structure methods to evaluate genetic changes and compared these to sequence-based methods. Overall, comparison among germline databases and sequence-based methods (CADD, Revel, SIFT, Fathmm, Lrt, and Metalr) showed many conflicting annotation results across the six proteins (**Figure 5A**). In contrast, our 3D approach, which leveraged 3D structures of the proteins encoded by these six genes, a level of information that is absent from nearly all clinical genomics studies, was able to add information about the spatial pattern of the alleles, suggesting common mechanisms for many (**Figure 5B**). Therefore, the 3D approach enhanced our ability to interpret genetic variants and prioritize further research for these six genes.

Among these six genes, we first assessed the gene with the most variants in our cohort (367 in total and 77 coding; **Figure 3C** and **Supplemental Figure S3C**), and the majority categorized as VUS (**Supplemental Table S5**). Across sequence-based algorithms, missense, splice-altering, and other LoF variants occurred throughout the protein sequence with no clear clusters of alterations (**Figure 5Ai**). In 3D, we identified a non-randomly distributed pattern where variants were located at the ends of helices from multiple domains and on the molecular surface (**Figure 5Bi**). This pattern is unusual and suggestive of a related effect across many variants *via* ATM surfaces involved in intermolecular interactions. This “helix capping” pattern is striking and suggests that a domain-centric approach to interpreting their effect would be insufficient. For instance, ATM has a domain that is critical for telomere maintenance and DNA damage repair (highlighted in pink, **Figure 5Bi**). Variants were recurrently observed at the base of this important domain. We suspect that the recurrent nature of these variants, combined with their spatial pattern, increases their likelihood for impacting ATM structure or function. We next investigated CHEK2, for which sequence-based methods commonly classified variants as deleterious (**Figure 5Aii**). Our use of 3D methods illustrated that most alleles in the protein encoded by *CHEK2* occur in the core of the kinase domain (**Figure 5Bii**), where they likely alter protein folding. Two variants impact the forkhead-associated (FHA) domain, which normally acts to bind phosphoserine motifs. Disruption of this functionally important region is likely to dysregulate signaling. Thus, we conjecture that these variants in the middle of the FHA domain (blue color in **Figure 5Bii**) may alter interactions with kinases that bind to CHEK2 for its regulation. Subsequently, we modeled MutSα, a heterodimeric enzyme formed by MSH2 and MSH6 and involved in DNA mismatch repair. Consequently, loss or decreased activity of either gene may be enough to impair enzymatic function (70, 71). In our cohort, coding variants in *MSH2* and *MSH6* were mutually exclusive, with 14 and 31, respectively, or 45 in total (**Figure 5Aiii**; **Supplemental Table S5**). In this case, sequence-based tools were consistent with each other in predicting alleles as either tolerated or damaging (**Figure 5Aiii**). The 3D enzyme has two channels (47). One channel, comprised of domains I, III, and IV, binds the mismatched DNA, while the other forms an ATPase domain, using residues from the domain V of both proteins. In 3D, the variants observed in our cohort occur throughout the structure, but primarily surround the ATP binding sites, rather than at the DNA interaction surface (**Figure 5Biii**). For example, MSH2 E809K is predicted to be tolerated by sequence-based methods, but in 3D is in close proximity to G322D, which has variable predictions by sequence-based scores. The 3D relationship among variants suggests a common effect on binding or ATPase activity. Additionally, there were several variants in the ATPase pore that have variable sequence-based predictions, such as MSH2 K603R and MSH6 L601H, which are nearby each other in 3D to support the potential of similar effects. These results reveal that 3D representation of mutational events extend the amount of evidence that can be used to interpret MutSα variants. PALB2 missense alleles were almost unanimously predicted to be tolerated by sequence-based algorithms (**Figure 5Aiv**). The 3D domain of PALB2 forms a radially symmetric 7-prop propeller fold (**Figure 5Biv**). Variants occur throughout the 3D structure. Because it is a symmetric molecule, we structurally aligned all 7 props and identified that the variants appear at the ends of the beta-strands (rotated and overlaid on right, **Figure 5Biv**). Moreover, four of the seven positions make inter-strand hydrogen bonds for which disruption could alter the conformation of the β-sheet. Further, one of the altered amino acids is a glycine and one a proline, which have unique backbone geometry compared to other amino acids, and three variants change small hydrophobic residues to larger hydrophilic residues. All five of these mutations could further disrupt the β-sheet and change the position of the adjoining loops, which likely influence how PALB2 interacts with other proteins. Finally, genetic variants in POLE, like MutSα, were predicted by sequence-based methods to be primarily tolerated (**Figure 5Av**). In 3D, POLE variants primarily occur at three regions, the interface between the exonuclease and the polymerase domains (**Figure 5Bv**), the helical bundle that defines the HS homology region (**Figure 5Bvi**), and along the periphery of the Domain of Unknown Function (DUF1744; **Figure 5Bvi**). This finding indicates that there may be different functions affected by alteration of each region, which is more evident by the 3D proximity of the alleles observed in our cohort than their position in the linear arrangement commonly used to described genes and protein sequences. In summary, the combination of sequence-based and 3D data suggests mechanisms by which variants are predicted to be damaging. Existing sequence-based methods cannot predict 3D contacts, bond disruptions, and the spatial relationships between functional sites. We believe that more comprehensive methods that leverage additional information, not predictable from the linear sequence, are needed to improve interpretation of genetic data. Moreover, our results support that potential mechanisms of protein dysfunction can be inferred in greater detail when assessing variants in 3D. These inferred mechanisms can then inform the design of specific future studies that seek to perform functional validation using experimental approaches. We envision that future studies from our group, using improved 3D methods to identify or prioritize the likely disease relevance of VUS (67, 68) will have an important impact on defining the mutational landscape underlying PDAC predisposition, development, and progression.

### Genetic Variants Identified in this Study Associate With Distinct Demographics, Serum Markers, and Clinical Outcomes

**Figure 6:**
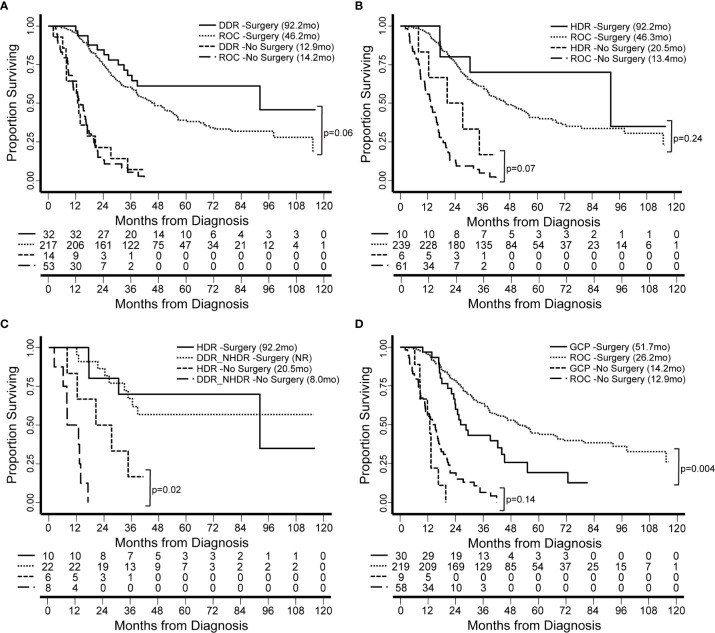
Patient survival is associated with genomics-based features. We identified groups of patients by presence of germline genetic features in specific pathways. Groups were compared based on median overall survival (OS, in months) from the date of diagnosis and for patients with resectable and borderline resectable PDAC. **(A)** Patients with pathogenic mutations in any DDR gene and who underwent surgery had longer OS than those with no DDR mutation (Rest of Cohort, ROC). **(B)** Focusing on the pathway of homology-directed repair (HDR), the same trend was observed for both patients that underwent and those that did not undergo surgery. **(C)** We compared patients with HDR mutations to those with other DDR mutations outside the HDR pathway (DDR_NHDR). We found that those who did not undergo surgery but had HDR mutations had longer OS. OS for DDR_NHDR-Surgery was not reached (NR). **(D)** We defined a genomic group by patients with germline alterations in genes related to genetic chronic pancreatitis (GCP). Patients in the GCP group had significantly shorter OS compared to those with no GCP alteration. Significant differences and p-values are indicated on the graphs.

We have thus far investigated patterns of germline alleles with a broad bioinformatic approach. In our final analysis, we synthesized our data to create groups of patients within our cohort based on genomics and tested the hypothesis that these groups carried associations with clinical features or clinical outcomes. We defined four groups based on DDR pathways and extent of surgical intervention (**Figure 6**). Groups were compared based on median overall survival (OS) from the date of diagnosis and for patients with resectable and borderline resectable PDAC. Patients with pathogenic mutations in any DDR pathway gene and who underwent surgery had longer OS than those with no DDR mutation (92.2 versus 46.2 months, p=0.06; **Figure 6A** **;** **Supplemental Table S9**). Focusing on the pathway of homology-directed repair (HDR), the same trend of better OS with a pathogenic mutation was observed for both patients that underwent surgery (92.2 versus 46.3 months; p=0.24) and those that did not undergo surgery (20.5 versus 13.4 months, p=0.07; **Figure 6B** **;** **Supplemental Table S10**). We compared patients with HDR mutations to those with other DDR mutations outside the HDR pathway (DDR_NHDR) and found that those who did not undergo surgery but had HDR mutations had longer OS (20.5 versus 8.0 months, p=0.02; **Figure 6C** **;** **Supplemental Table S11**). We defined a genomic group by patients with germline alterations in genes related to chronic pancreatitis (CP), which we will refer to as genetic chronic pancreatitis (GCP). Patients in the GCP group had significantly shorter OS compared to those with no GCP alteration, when they underwent surgery (51.7 versus 26.2 months, p=0.004; **Figure 6D**). Therefore, we also tested OS using univariate and multivariate models accounting for race, preoperative CA19-9, and stage. Univariate models indicated high mortality associated with being in the GCP group (hazard ratio, HR=1.9; p=0.005), while the multivariate model had an attenuated effect (HR = 1.5; p = 0.13). GCP was linked with patient race, with a higher proportion of non-white individuals in the GCP group (p < 0.001; **Supplemental Table S12**); further study is needed to understand the independent contributions of these two factors. Thus, the functional impact of mutations in GCP-associated genes on PDAC outcomes, even among patients who do not present with CP, warrants further study.

Next, we defined additional patient groups based solely on germline genetic patterns (**Table 4**) such as the presence of pathogenic alleles, pathways, and those associated with heritable cancer risk in previous germline studies. In this manner, we defined 18 groups (**Table 4**, **Supplemental Figure S5**), coded them by numbers 1–18, and correlated each of these groups with clinical characteristics and OS. We found that among patients who were producers of CA19-9, those that had any pathogenic variant (67 patients, 12.5%) had higher pre-treatment CA19-9 levels (median of 498 U/ml (n=44) versus 293 (n=259); p = 0.02). Among patients with any pathogenic allele and metastatic disease, we observed better OS compared to the rest of the cohort (22.0 months (n=10) versus 9.8 (n=52); p = 0.008). When the pathogenic variants were previously reported for heritable cancer syndromes, patients were younger (58 years (n=20) versus 66 (n=515); p = 0.02); the further subset of patients with metastatic disease had better OS compared to the rest of the cohort (23.2 months (n=5) versus 9.9 (n=57); p = 0.01). Thus, there may be divisions among pathogenic variants that have different clinical ramifications for PDAC.

**Table 4:** Genomics-based patient groups differ in clinical features and outcomes.

Group Identifier	Group Description	Genomic Features			Size of Group (# with follow up)	Clinical Measure	Value for:		p-value
		Pathogenic	Germline versus Somatic Resource^†^	Gene(s)^‡^			Patients in Group	Rest of Cohort	
Pathogenic	Any pathogenic variant	Yes	G or S		67 (60)	Pre-treatment CA19-9, U/ml (median)	498	293	0.02
						Survival among Metastatic (months)	22	9.8	0.008
						Survival among Metastatic (months)	10.9	9.9	0.04
Population Enriched 10x	Any variant that is enriched by 10x in our cohort compared to gnomAD		G or S	MAF ≥ 10x	205 (179)	(n.s.)			
Cancer VUS	VUS reported in somatic or germline databases, with a cancer phenotype		G^C^ or S		467 (403)	Patient Age	65	68	0.002
Cancer Either Pathogenic	Pathogenic variants from either germline or somatic databases	Yes	G^C^ or S		42 (39)	Survival among Metastatic (months)	16.2	9.8	0.03
Genetic Cancer	Germline variants with a cancer or cancer risk phenotype, that are not reported in somatic databases		G^C^ and not S		250 (219)	Race			0.002
						Milwaukee county resident	63	49	0.02
Genetic Cancer Pathogenic	Pathogenic variants from germline databases, that are not reported in somatic databases	Yes	G^C^ and not S		20 (20)	Age	58	66	0.002
						Survival among Metastatic (months)	23.2	9.9	0.01
Somatic Cancer	Somatic variants that are not reported in a germline database		S and not G^C^		124 (109)	Patient Race			0.01
						Survival among Metastatic (months)	12.2	9.7	0.003
Somatic Cancer Pathogenic	Pathogenic variants observed in somatic databases, but not in germline databases for a cancer phenotype	Yes	S and not G^C^		11 (10)	(n.s.)			
Genetic, Somatic Cancer	Any variant reported in both somatic and germline databases, with a cancer phenotype		G^C^ and S		401 (346)	(n.s.)			
Genetic, Somatic Cancer Pathogenic	Pathogenic variants reported in both germline and somatic databases	Yes	G^C^ and S		11 (9)	Pre-treatment CA19-9, U/ml (median)	598	302	0.03
						Survival among resectable (months)	17.1	50.6	0.01
PDAC Cancer	Any somatic variant observed in pancreatic cancer samples		S for PDAC		64 (53)	(n.s.)			
ATM	Any variant in ATM		G or S	ATM	142 (126)	Race			< 0.001
						Radiographic Stage			0.04
ATM Pathogenic	Pathogenic variants in ATM	Yes	G or S	ATM	5 (4)	Vital Status			0.05
BRCA1/BRCA2	Any variant in BRCA1 or BRCA2		G or S	BRCA1, BRCA2	244 (202)	Survival among resectable (months)	79.4	38.9	0.05
BRCA1/BRCA2 Pathogenic	Pathogenic variants in BRCA1 or BRCA2	Yes	G or S	BRCA1, BRCA2	7 (7)	(n.s.)			
HDR	Any variant in an HDR gene		G or S	HDR	342 (290)	(n.s.)			
HDR Pathogenic	Pathogenic variants in HDR genes	Yes	G or S	HDR	16 (15)	Survival among locally advanced (months)	not reached	29.1	< 0.001
Germline Cancer Risk	Germline variants from ClinVar or HGMD with a cancer risk phenotype		G^C^		155 (135)	(n.s.)			

^†^G, variant observed in a germline resource; S, variant observed in a somatic resource. G^C^, the subset of germline variants which are reported for a cancer or cancer risk phenotype.

^‡^Variants present in the listed gene symbols. HDR indicates the group of genes responsible for homology-directed repair. MAF ≥ 10x indicates an enrichment of any variant in our cohort, relative to gnomAD, by a factor of 10 or more.

n.s., no significant associations.

The same set of clinical features and outcomes were tested for each group; only significant associations are shown here.

Patients in our cohort who had variants previously identified from somatic studies, were more likely to be non-white (18% versus 9%; p = 0.01). Interestingly, patients in this group who also had metastatic disease, showed a better OS (12.2 months (n=20) versus 9.7 (n=42); p = 0.003). When the variant was also previously associated with an inherited disease, those who were producers of CA19-9 had higher pre-treatment levels of CA19-9 (598 U/ml (n=8) versus 302 (n=295); p = 0.03); the further subset with resectable disease had poorer OS (17.1 months (n=3) versus 50.6 (n=151); p = 0.01; independent of neoadjuvant treatment). Thus, the interpretation of germline genetic variation underlying PDAC should be informed by knowledge from somatic profiling.

We tested groups defined by genetic alteration of key PDAC genes. Patients with variants in *ATM* were associated with non-white origin (p < 0.001), and had a lower frequency of locally advanced disease. Instead, they had a higher frequency of metastatic disease (p = 0.04), concordant with previous reports in brain and breast cancers (72, 73); 15.6% (n=84) of our cohort had metastatic disease and 20.2% (n=109) had locally advanced. Patients with any *BRCA1* or *BRCA2* variants and resectable disease had better OS (79.4 months (n=68) versus 38.9 (n=86); p = 0.05). We found additional trends among groups defined by pathogenic variants in *BRCA1*, *BRCA2*, and other DDR pathway genes, but due to the few cases available, the associations were often not statistically significant but should be considered when planning larger studies (**Table 4**). Further and more detailed descriptions of these associations between genomics-based groups with clinical features and outcomes are available in **Supplemental Text** and **Table 4**. We have found that the germline genomic findings within our cohort associate with clinical outcomes, thereby indicating that our broader bioinformatic approach has the potential to improve the practice of precision medicine, such as for family counseling of PDAC-affected patients in referral populations.

## Discussion

Advances in the field of genetics have extended our understanding of how genomic variation in the germline predisposes to cancer, such as the subset of well-known cancer genes including *BRCA1*, *BRCA2*, *TP53*, *CDKN2A*, *ATR*, and *ATM (* 2, 3, 18, 19). Our study sought to identify groups of patients, previously unrecognized, that have genetic variants that may be modifiers of their risk for development of PDAC or clinical and pathobiology course. While alteration of the genes mentioned above is known to influence the development of many types of malignancies, all patients in our cohort have at least one genetic variant that currently lacks medical interpretation (VUS) in well-established cancer genes, potentially limiting our understanding of germline genetic contributions to PDAC. We have described: 1. The design and use of an exon panel that contained previously tested and newly identified genes with the potential to influence cancer development; 2. The reported incidence and type of genomic variation captured by our panel at the cohort, gene, and patient level; 3. The development of an enhanced annotation approach based on using genomic variation data previously found in both germline and somatic diseases; 4. The combined use of both sequence-based and 3D bioinformatics methods for classifying variants; and, 5. The associations among our genomic results, clinical disease characteristics, and patient outcomes. Our findings confirm large genomic variation, either represented by clear pathogenic variants or those of uncertain significance (**Figure 1A**, **Table 2**), in genes with known pathobiological roles in PDAC. Thus, it is important to discuss the significance of this variation and draw inferences of their relevance to pancreatic cancer to improve quantification of genetic risk.

We designed our gene panel to focus on biologic pathways with known biologic relevance to pancreatic cancer, such as DNA Damage response, cell cycle checkpoints, and cell adhesion and migration. In addition, we explored genes associated with chronic pancreatitis for which there is growing evidence to indicate a relationship to the development of PDAC. Overall, we found a higher level of pathogenic variants than in previous studies (2, 3, 18, 19) which could be due to the composition of our gene panel or specific characteristic of our patient population, referred from a broad geographic sampling of this country and varied in race and ethnicity. For example, all patients in our cohort had a VUS in BRCA2, highlighting that there may be many un-tested alleles within this gene despite the existence of medium-throughput cellular studies that have been used to characterize many observed alleles (63, 74). Further, BRCA2 is one of the genes involved in double-strand break repair and the other members of the pathway have received less scrutiny even though they are also recurrently altered in human cancers. Therefore, we believe the description of germline PDAC-predisposing variation is incomplete.

The potentially incomplete description begs an important scientific question as to the functional differences between an allele present in an individual *via* germline or somatic alteration. To address this question, we used a more integrated approach to genomic annotation, enabling us to suggest which of the many observed VUS were more likely than others to bear functional relevance to our patient’s disease. For example, we found that a third of the variants we found have been previously reported, and of those, 26% were previously also observed in tumor samples. These somatic VUS, which were observed in our patient’s germline, bear a greater level of evidence for being related to cancer, compared to VUS not previously observed, or found in the germline setting. We tested the association between somatic VUS and patient outcomes, although they were confounded by patient race (non-white patients were more likely to have somatic VUS) indicating that greater study is required to understand the independent effects of genetic and other factors. Observation of somatic VUS is important since some, though not all (e.g., CFTR), pancreatic cancer predispositions genes, could come from families whose members may develop other type of tumors.

We also provide information that reveals the relationship of the VUS to other germline diseases, findings that are often absent from oncology reports. This is important to allow investigators and clinicians to be aware of pathogenic variants which may increase the risk for PDAC. For example, there are patients with pathogenic *ATM* alleles, which are known to cause the congenital disease ataxia-telangiectasia (ATS), which also convey an increased risk of developing cancer (75–78). Thus, ATS alleles, even among patients who do not have ATS, may yet bear significance for PDAC risk. We also observe a significant number of CFTR (79–81), CTRC (82), and CPA1 (82, 83) variants, which supports the association between chronic pancreatitis and PDAC risk. Finally, patients with DDR alterations had longer OS than other patients, and we identified numerous associations among specific cellular pathways and OS. Thus, our enhanced annotation approach extended the mechanistic and medially relevant information that could be derived from the genomic data.

Additionally, inferences on the potential damaging and mechanistic effects of variants on the encoded 3D protein, rather than the genomic DNA, are important but less considered in translational genomics. Our laboratory routinely uses this type of methodology for our study of inherited diseases (68, 69, 84–86) and, similar to what is shown here, the encoded protein itself added more information of potential mechanisms of dysfunction due to genome variants. We believe that lessons learned from biophysics and protein science have potential to aid the interpretation of genomic variation, evidenced by our identification of spatial patterns among the VUS observed in our PDAC patients. These patterns enabled us to make hypotheses about the functional mechanism affected by each VUS. Hence, this study adds new genetic information of potentially medical significance by describing genomic patterns and mechanistic hypotheses that can be the subject of future study.

Because of the normal role of the pancreas in digestion, there has been long interest in the potential link between diet and disease. However, dietary effects variable between studies; their clear interpretation remains to be established (89–91). Thus, future studies could be designed to characterize interactions among the genetic associations we report herein and modifiable factors including diet, and their combined influence on cancer and pre-cancer states. The additional concept of genetic pancreatitis – a pro-neoplastic genetic predisposition related to an established disease, but not identified as the disease – may be critical for harmonizing different effects that are currently considered variable into a common model, possibly through their shared dysregulation or over-exertion of normal pancreatic function.

In conclusion, we present data that indicates a strong relationship between germline alleles, functional mechanisms, and patient outcomes, that improve our understanding of the etiology and progression of PDAC. Relationships between patient race, genetic variants, heritable and chronic diseases, and outcomes must be deconvoluted. We believe that the understanding of germline genetic contributions to PDAC is incomplete, but through the broader study of multiple tiers of information, a more complete functional understanding will be achieved.

## Data Availability Statement

All genetic variants discussed in detail and underlying our findings, the minimal data set, are reported in this study. Sharing full genomic sequencing of our patient germline samples was not IRB approved, and thus is only available through future IRB-approved collaborative agreements.

## Ethics Statement

The studies involving human participants were reviewed and approved by Medical College of Wisconsin (MCW) IRB and used the Surgical Oncology Tissue Bank (PRO # 12151), which provided the samples, and the Pancreatic Cancer Clinical Database (PRO # 12479), which provided all clinical data. The patients/participants provided their written informed consent to participate in this study.

## Author Contributions

MZ, GL, DE, and RU conceived and designed the study. DE and ST provided access to samples. AM and MT performed sample processing and high-throughput sequencing. MZ, TS, JA, WD, and AM performed bioinformatic analyses and generated figures. MA and ST performed survival analysis and generated figures; MZ and DE contributed to survival analysis design. MZ, AM, and RU wrote the manuscript. All authors contributed to the article and approved the submitted version.

## Funding

This work was supported by the Theodore W. Batterman Family Foundation, Inc., the MCW Department of Surgery, the National Institutes of Health Grants R01 CA178627 (to GL) and R01 DK52913 (to RU), the Advancing a Healthier Wisconsin Endowment (to each GL and RU), the Linda T. and John A. Mellowes Endowed Innovation and Discovery Fund (to RU), the Ronald Burklund Eich Pancreatic Cancer Tissue Bank Fund (to ST, MA, and DE), which supports the Surgical Oncology Tissue Bank, the Medical College of Wisconsin LaBahn Pancreatic Cancer Program and the We Care Fund for Innovation and Discovery in the Department of Surgery (to ST, MA, and DE).

## Conflict of Interest

The authors declare that the research was conducted in the absence of any commercial or financial relationships that could be construed as a potential conflict of interest
