# Supplementary material for: Interpreting Sequence Variation in PDAC-Predisposing Genes Using a Multi-Tier Annotation Approach Performed at the Gene, Patient, and Cohort Level
Source: Front Oncol. 2021 Mar 5;11:606820. doi: 10.3389/fonc.2021.606820 (PMC7973372; doi:10.3389/fonc.2021.606820)
Supplement: Supplementary file 1 [file DataSheet_1.docx]

Interpreting Sequence Variation in PDAC-Predisposing Genes Using a Multi-Tier Annotation Approach Performed at the Gene, Patient and Cohort Level

Michael T. Zimmermann^1,2,3,*^, Angela J. Mathison^4,5^, Tim Stodola^4^, Douglas B. Evans^6,9^, Jenica L. Abrudan^5^, Wendy Demos^5^, Michael Tschannen^5^, Mohammed Aldakkak^6^, Jennifer Geurts^5,7^, Gwen Lomberk^4,5,9^, Susan Tsai^4,6,9^ and Raul Urrutia^3,4,5,9,*^

^1^Bioinformatics Research and Development Laboratory, Genomic Sciences and Precision Medicine Center, Medical College of Wisconsin, Milwaukee, WI 53226, USA

^2^Clinical and Translational Sciences Institute, Medical College of Wisconsin, Milwaukee, WI 53226, USA

^3^Department of Biochemistry, Medical College of Wisconsin, Milwaukee, WI 53226, USA

^4^Division of Research, Department of Surgery, Medical College of Wisconsin, Milwaukee, WI 53226, USA

^5^Genomic Sciences and Precision Medicine Center, Medical College of Wisconsin, Milwaukee, WI 53226, USA

^6^Division of Surgical Oncology, Department of Surgery, Medical College of Wisconsin, Milwaukee, WI 53226, USA

^7^Genetic Counseling Program, Medical College of Wisconsin, Milwaukee, WI 53226, USA

^8^Department of Pharmacology and Toxicology, Medical College of Wisconsin, Milwaukee, WI 53226, USA

^9^LaBahn Pancreatic Cancer Program, Medical College of Wisconsin, Milwaukee, WI 53226, USA

^*^Corresponding Author:

Electronic Address: mtzimmermann@mcw.edu rurrutia@mcw.edu

Telephone: Office: 414-955-8550

Administration: 414-955-4887

Mailing Address: Genomic Sciences and Precision Medicine Center (GSPMC)

Human Research Center, 5th Floor

Medical College of Wisconsin

8701 Watertown Plank Road

Milwaukee, Wl 53226-0509

**Keywords:** pancreatic cancer, genetic predisposition, genomics, medical oncology, genomic data interpretation, precision oncology, germline-somatic interaction

# Supplemental Text

Genomic Variant Call Quality

Our paradigm in this study was to assess the potential for genetic variation in the germline to have an impact on cancer likelihood or etiology, and to build the case for which genes require improved methods for interpreting genetic variants.

We assessed DNA variant quality using multiple parameters including: read depths, B-allele frequency, cohort-level genotype quality, and sample-level genotype quality.

We identified all variants that passed minimal technical QC thresholds using the following criteria: cohort variant quality > 20, per-sample variant quality in any sample > 10, per-sample alternate allele depth in any sample > 4, per-sample BAF in any sample > 15%. We then assessed QC in more detail. There were 5 pathogenic variants that would have been missed with more strict filtering. Thus, to be comprehensive for pathogenic allele detection, we maintained all variants.

Our analyses focused on coding more than on non-coding variants and coding variants had significantly fewer variants of suspicious quality compared to non-coding variants.

Quality of Amplicons in Variant Classes

|  | **benign** | **somatic VUS** | **other** | **novel VUS** | **reported VUS** | **pathogenic** |
| --- | --- | --- | --- | --- | --- | --- |
| **Suspicious Quality** | 33 | 9 | 1 | 1816 | 13 | 6 |
| **Good Quality** | 1068 | 25 | 7 | 2137 | 777 | 69 |

Technical Quality of Coding versus Non-Coding Variants

|  |  | **Coding** | |
| --- | --- | --- | --- |
|  |  | **No** | **Yes** |
| **Suspicious Quality** | **No** | 1899 | 124 |
|  | **Yes** | 3214 | 869 |

Pathogenic Variants with Suspicious Quality

| **gene** | **variant2** | **hgvs.p** | **clinvar** | **HGMD** | **cosmicS1** | **tcga** | **ClinVar Info** |
| --- | --- | --- | --- | --- | --- | --- | --- |
| *ATM* | 11:108119772_G/A | p.Trp393* | TRUE | FALSE | . | FALSE | Pathogenic with single submitter (Ambry) |
| *BRCA2* | 13:32911442_GA/G | p.Asn986fs | TRUE | TRUE | . | FALSE | NotPresent? |
| *MSH6* | 2:48010633_G/C |  | TRUE | FALSE | . | FALSE | LP with single submitter (Ambry) |
| *RAD50* | 5:131931451_TA/T | p.Lys583fs | TRUE | FALSE | AC | FALSE | Not Present? |
| *PMS2* | 7:6045569_TA/T | p.Val39fs | TRUE | FALSE | . | FALSE | Pathogenic with single submitter (GeneDX) |

*BRCA2*: The neighboring Asn has a N987fsdeletion that is ***Pathogenic

*MSH6*: splice-alteration

*RAD50*: AC:Adenocarcinoma, Overlaps NM_005732.3(RAD50):c.2156dupT(p.Glu723Glyfs*5) with **Pathogenic

Clinical Characteristics and Outcomes Associations with Genomics-Based Groups

We first tested genomic groups defined by pathogenic alleles. We found that among patients who were producers of CA19-9, those that had any pathogenic variant (67 patients, 12.5%) had higher pre-treatment CA19-9 levels (median of 498 U/ml (n=44) versus 293 (n=259); p = 0.02). Among patients with any pathogenic allele and metastatic disease, we observed better OS compared to the rest of the cohort (22.0 months (n=10) versus 9.8 (n=52); p = 0.008). When the pathogenic variants were previously reported for heritable cancer syndromes, patients were younger (58 years (n=20) versus 66 (n=515); p = 0.02); the further subset of patients with metastatic disease had better OS compared to the rest of the cohort (23.2 months (n=5) versus 9.9 (n=57); p = 0.01). Thus, there may be divisions among pathogenic variants that have different clinical ramifications for PDAC.

Patients in our cohort who had germline variants previously identified from somatic studies, were more likely to be non-white (18% versus 9%; p = 0.01). Interestingly, patients in this group who also had metastatic disease, showed a better OS (12.2 months (n=20) versus 9.7 (n=42); p = 0.003). When patient samples had pathogenic alleles, those who were producers of CA19-9 had higher pre-treatment levels of CA19-9 (598 U/ml (n=8) versus 302 (n=295); p = 0.03); the further subset with resectable disease had poorer OS (17.1 months (n=3) versus 50.6 (n=151); p = 0.01; independent of neoadjuvant treatment).

We tested groups defined by genetic alteration of key PDAC genes. Patients with variants in *ATM* were associated with non-white origin (p < 0.001), and less often had locally advanced disease. Instead, they had higher metastatic burden (p = 0.04). Patients with any *BRCA1* or *BRCA2* variants and resectable disease had better OS (79.4 months (n=68) versus 38.9 (n=86); p = 0.05; the further subset receiving neoadjuvant therapy: 55.1 months versus 38.9; p = 0.17), which indicates that some of the VUS are likely to be damaging and should be considered in future studies. . We examined outcomes for patients with variants in any genes responsible for homology-directed repair to investigate the strength of OS signal from VUS in these genes, and found no statistically significant differences, despite better OS for resectable disease (55.1 months (n=96) versus 38.6 (n=58); p = 0.19; the further subset receiving neoadjuvant therapy: 43.6 months vs. 38.9 months; p = 0.42)), and worse for metastatic patients (9.1 months (n=44) versus 13.3 (n=18); p = 0.13). Therefore, specific genes and PDAC-relevant pathways carry information with clinical relevance, for pathogenic and uncertain alleles, indicating that alleles currently regarded as VUS are likely to be functional. In conclusion, the relationship of variants-to-race-outcome was unexpected but, not surprising, when considering that our institution is located within a city consisting of a higher percentage of non-white inhabitants.

# Supplemental Figures


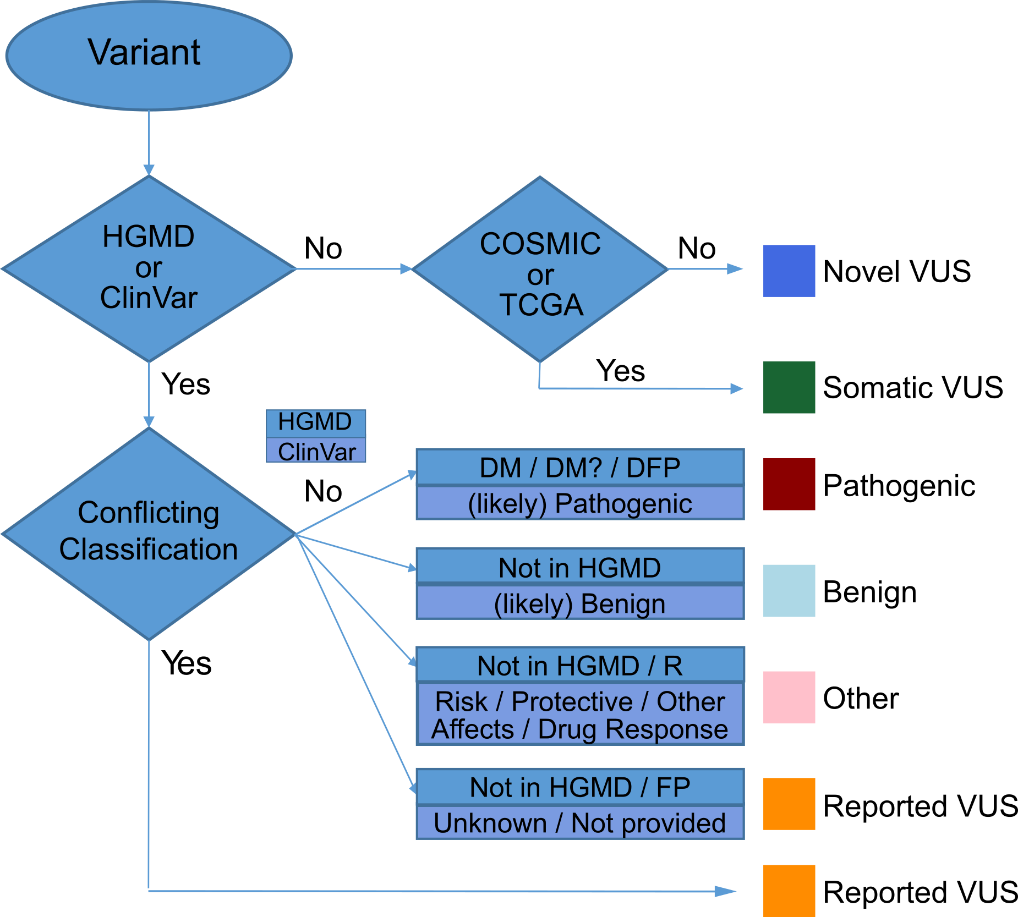


**Supplemental Figure S1: Classification algorithm used to categorize all variants with four databases: HGMD, ClinVar, COSMIC, and TCGA.** For variants with conflicting classification, we distinguish terms from HGMD (blue boxes) and ClinVar (purple boxes). Classification abbreviations in HGMD include: disease causing mutation (DM), likely disease-causing mutation (DM?), disease-associated polymorphisms with supporting functional evidence (DFP), retired (R), functional polymorphism (FP), and disease associated polymorphism (DP).

**
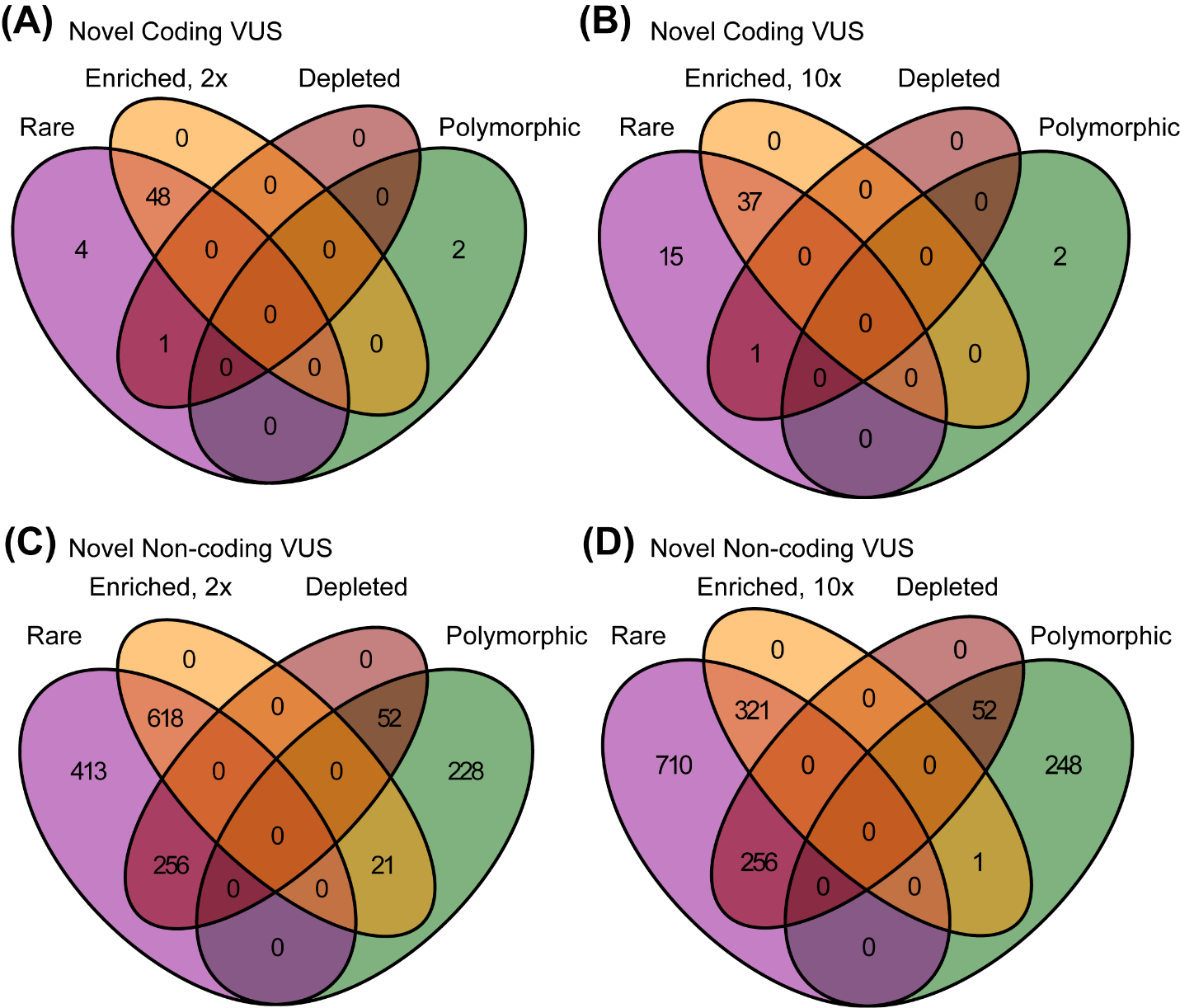
**

**Supplemental Figure S2: Novel VUS are Rare relative to the general population. (A)** The distribution of novel coding VUS by population frequency, grouped into rare (>0% and <5% in gnomAD) versus polymorphic (≥5% in gnomAD) and the ratio of cohort to population frequency (enriched at a ratio ≥2 versus depleted at a ratio <0.5). **(B)** Adjusting the enrichment to a ratio ≥10. **(C)** The distribution of novel non-coding VUS by population frequency (rare versus polymorphic) and the ratio of cohort to population frequency (enriched at a ratio ≥2 versus depleted at a ratio <0.5). **(D)** Adjusting the enrichment to a ratio ≥10. Only variants present in gnomAD database were utilized for these calculations.

**
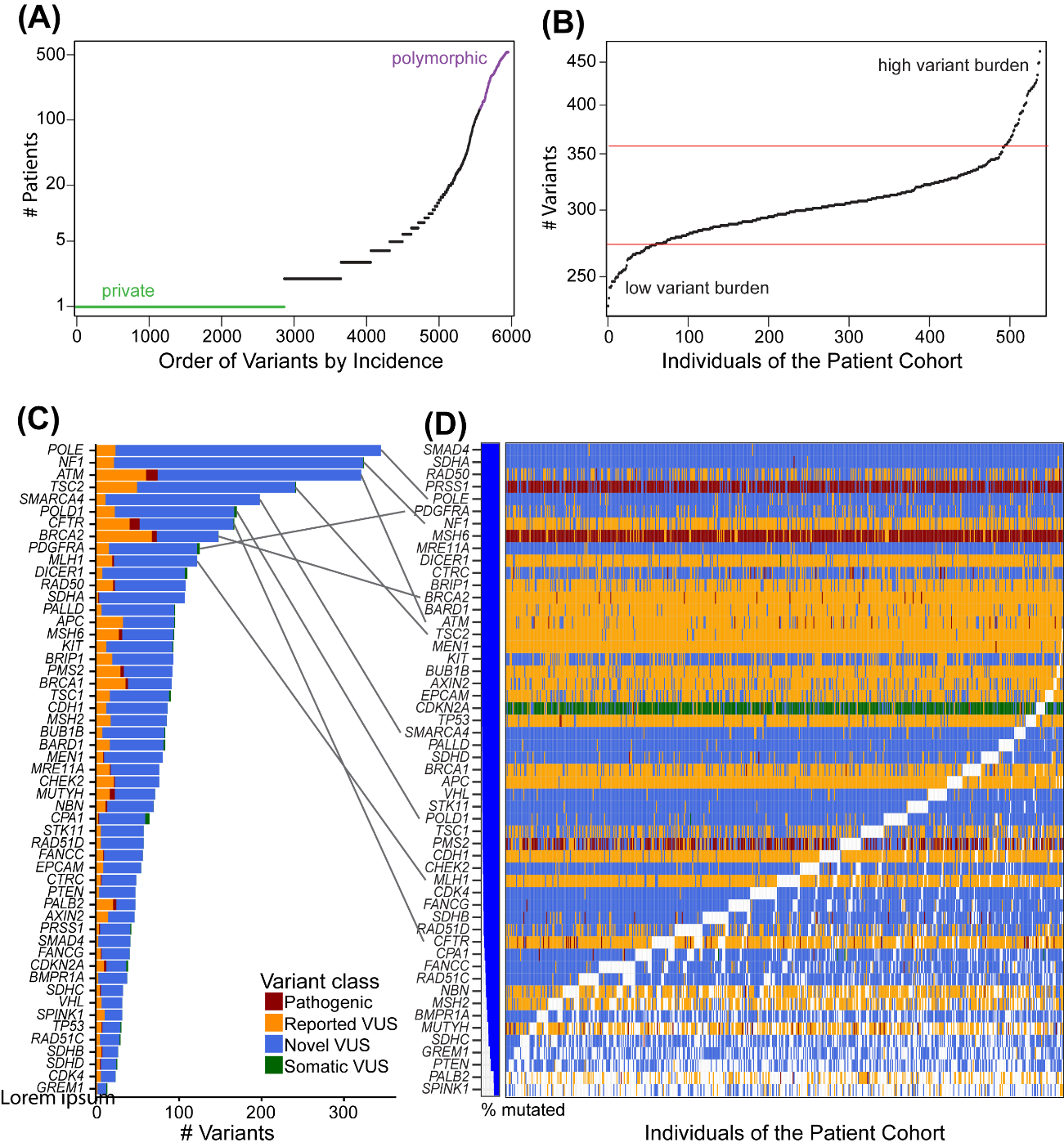
**

**Supplemental Figure S3: Distribution and quantification of variants in the patient cohort. (A)** Ordering all variants by incidence number in patients with a distribution from private mutations (green) to polymorphic (purple) alleles. **(B)** The number of all variants determined on a per patient basis. **(C)** Ordered list of all unique variants, further subdivided by classification type, for each gene. **(D)** Incidence of all variants across the cohort of patients showed a different order of genes (see gray connections for top 10 genes in **(C)**). When multiple variants of a gene are present in a single patient the color representation was indicated in the order of pathogenic, reported VUS, and then novel VUS.


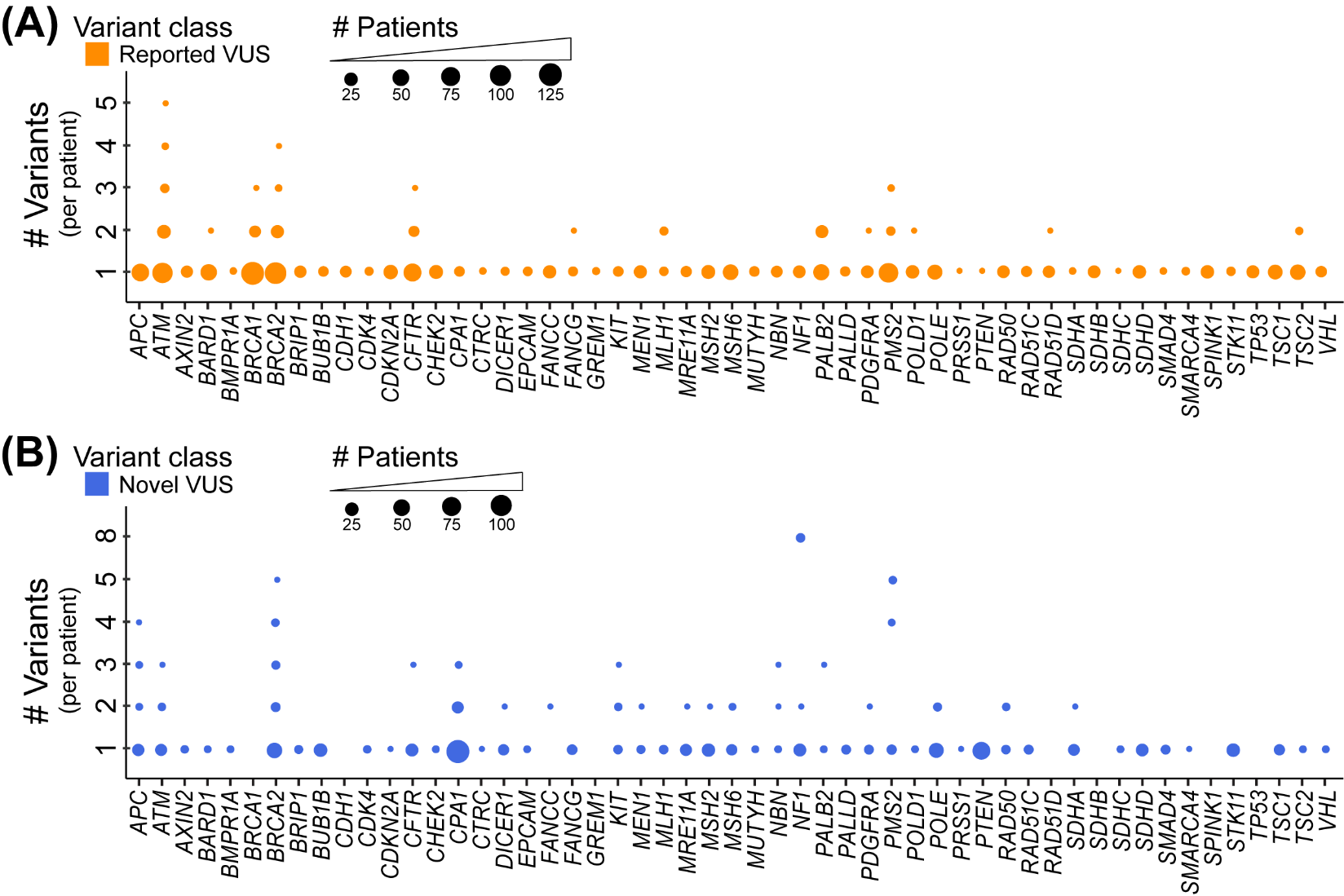


**Supplemental Figure S4: Multiple variants per gene are observed per patient in the PDAC panel.** Bubble plots with additional subdivision of the number of coding variants **(A)** reported VUS and **(B)** novel VUS that occur per patient per gene. The bubble size is proportional to the number of patients that have single or multiple variant(s) in a particular gene.


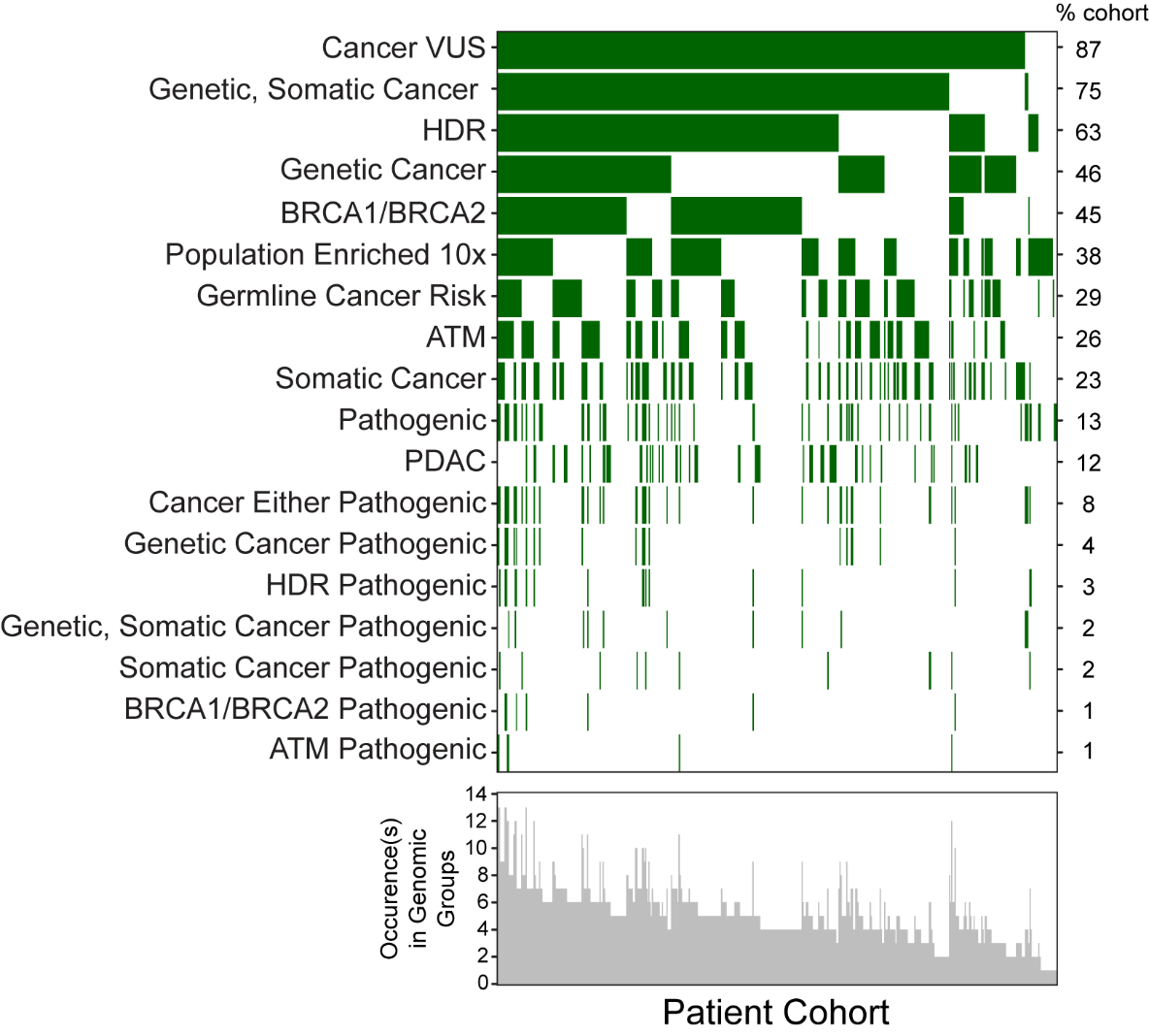


**Supplemental Figure S5: Genomics-based patient groups.** Each column of this heatmap represents a patient in our cohort. Each row is a group defined by genomic criteria. Patients are indicated as being present in a group by a green line and sorted in a waterfall order where all patients in the first group are ordered first, followed by those who are also in the second group, and so on. Groups/Rows are ordered by the fraction of the cohort that has the genomic criteria. Below the heatmap is a plot showing the number of genomic groups that each sample is included within.

# Supplemental Tables

Supplemental Tables are available in the attached Excel file. Each tab of the file has one supplemental table in the following order.

**Supplemental Table S1:** Median coverage of sequencing across all samples per gene

**Supplemental Table S2:** Somatic VUS, variants annotated in somatic disease only

**Supplemental Table S3:** Number of variants (coding and non-coding) identified in each gene sequenced.

**Supplemental Table S4:** Variant annotations based on subtypes of coding and non-coding effect ontology identified by SnpEff

**Supplemental Table S5:** Identification of 75 pathogenic variants in the germline sequence of the patient cohort

**Supplemental Table S6:** CFTR coding variants (non-benign) seen in more than 1 patient in our cohort

**Supplemental Table S7:** ATM coding variants (non-benign) seen in more than 1 patient in our cohort

**Supplemental Table S8:** BRCA2 coding variants (non-benign) seen in more than 1 patient in our cohort

**Supplemental Table S9:** DDR vs. Rest of Cohort

**Supplemental Table S10**: HDR vs. Rest of Cohort

**Supplemental Table S11:** DDR excluding HDR (DDR_NHDR) vs. HDR

**Supplemental Table S12:** GCP vs. Rest of Cohort
